# Diverse impact of xeno-free conditions on biological and regenerative properties of hUC-MSCs and their extracellular vesicles

**DOI:** 10.1007/s00109-016-1471-7

**Published:** 2016-09-16

**Authors:** Sylwia Bobis-Wozowicz, Katarzyna Kmiotek, Karolina Kania, Elzbieta Karnas, Anna Labedz-Maslowska, Malgorzata Sekula, Sylwia Kedracka-Krok, Jacek Kolcz, Dariusz Boruczkowski, Zbigniew Madeja, Ewa K. Zuba-Surma

**Affiliations:** 1 Department of Cell Biology, Faculty of Biochemistry, Biophysics, and Biotechnology, Jagiellonian University, 30-387 Krakow, Poland; 2 Malopolska Centre of Biotechnology, 30-387 Krakow, Poland; 3 Department of Physical Biochemistry, Faculty of Biochemistry, Biophysics, and Biotechnology, Jagiellonian University, 30-387 Krakow, Poland; 4 Department of Pediatric Cardiac Surgery, Polish-American Children’s Hospital, 30-663 Krakow, Poland; 5 Polish Stem Cell Bank, 00-131 Warsaw, Poland

**Keywords:** Extracellular vesicles, Mesenchymal stem cells, Umbilical cord, Xeno-free, Immunomodulation, Heart regeneration

## Abstract

**Abstract:**

Growing evidence indicates that intracellular signaling mediated by extracellular vesicles (EVs) released by stem cells plays a considerable role in triggering the regenerative program upon transplantation. EVs from umbilical cord mesenchymal stem cells (UC-MSC-EVs) have been shown to enhance tissue repair in animal models. However, translating such results into clinical practice requires optimized EV collection procedures devoid of animal-originating agents. Thus, in this study, we analyzed the influence of xeno-free expansion media on biological properties of UC-MSCs and UC-MSC-EVs for future applications in cardiac repair in humans. Our results show that proliferation, differentiation, phenotype stability, and cytokine secretion by UC-MSCs vary depending on the type of xeno-free media. Importantly, we found distinct molecular and functional properties of xeno-free UC-MSC-EVs including enhanced cardiomyogenic and angiogenic potential impacting on target cells, which may be explained by elevated concentration of several pro-cardiogenic and pro-angiogenic microRNA (miRNAs) present in the EVs. Our data also suggest predominantly low immunogenic capacity of certain xeno-free UC-MSC-EVs reflected by their inhibitory effect on proliferation of immune cells in vitro. Summarizing, conscious selection of cell culture conditions is required to harvest UC-MSC-EVs with the optimal desired properties including enhanced cardiac and angiogenic capacity, suitable for tissue regeneration.

**Key message:**

- Type of xeno-free media influences biological properties of UC-MSCs in vitro.
- Certain xeno-free media promote proliferation and differentiation ability of UC-MSCs.
- EVs collected from xeno-free cultures of UC-MSCs are biologically active.
- Xeno-free UC-MSC-EVs enhance cardiac and angiogenic potential of target cells.
- Type of xeno-free media determines immunomodulatory effects mediated by UC-MSC-EVs.

**Electronic supplementary material:**

The online version of this article (doi:10.1007/s00109-016-1471-7) contains supplementary material, which is available to authorized users.

## Introduction

The therapeutic potential of mesenchymal stem cells (MSCs) not only has been widely studied in animal models [1] but was also validated for human patients, indicating efficacy in the treatment of a variety of diseases [2, 3]. Among different populations of MSCs isolated from various sources, umbilical cord-derived MSC (UC-MSCs) are particularly promising, due to their high proliferative potential, longevity, immunomodulatory properties, and noninvasive isolation procedure [4]. Interestingly, although the beneficial effects of stem cell-based therapy have been widely appreciated, the level of engrafted cells was usually very low suggesting that the paracrine activity of stem cells rather than direct differentiation plays a key role in tissue repair [5]. Among paracrine factors, circular cell-membrane enclosed cytoplasmic structures termed extracellular vesicles (EVs) have been recognized as important mediators of cell-to-cell communication [6]. It was shown that EVs can interact with cell surface receptors or fuse with a cell membrane and transfer bioactive cargo in the form of messenger RNA (mRNA), microRNA (miRNA), proteins, and lipids to the recipient cells, triggering specific intracellular signaling pathways, thereby influencing cell fate [6, 7]. Importantly, compelling evidence supports the significance of EV-mediated signaling in a variety of physiological and pathological processes, including embryonic development, the maintenance of tissue homeostasis [8], spread of pathogens or modulating disease onset, and progression [9]. Prominently, EVs derived from human mesenchymal stem cells (MCS-EVs) were shown to enhance regeneration of various tissues in animal models [10–16], thus creating a new treatment option for many disorders. In particular, such therapy could be used to improve regeneration of the infarcted or diseased heart, conditions which are the leading causes of morbidity and mortality worldwide [17], despite the available modalities. To prove this theory, there are reports showing that MSC-EVs infusion to the experimental animals ameliorated myocardial ischemia/reperfusion injury [14] and markedly enhanced blood flow recovery accompanied by reduction of the infarct size of the heart [15]. However, to fully translate the beneficial effects of MSC-EVs treatment into the clinic, there is a need to carefully define and standardize cell culture conditions for EVs collection, devoid of any substrates of animal origin.

Various chemically defined xeno-free media dedicated to support MSCs growth and characteristics have been offered on the market and their ability to maintain MSCs properties has already been confirmed [18–21]. However, the impact of such media on the therapeutic properties of MSCs and MSC-derived EVs has never been studied. Since MSC-EVs may be an attractive cell substitute in future clinical applications, in this study, we provide, for the first time, a comprehensive analysis of the biological activities of UC-MSCs and their acellular derivatives such as EVs in five different xeno-free media, compared to a standard serum-based medium and a serum-reduced medium supplemented with basic fibroblast growth factor (bFGF). Our goal was to identify the most optimal serum-free and xeno-free cell culture conditions for collection of therapeutically valuable UC-MSC-EVs for their potential use in heart regeneration.

## Materials and methods

### Cell culture

Human UC-MSCs, human umbilical vein endothelial cells (HUVECs), human cardiac mesenchymal stromal cells (cMSCs), and human peripheral blood mononuclear cells (PBMCs) were obtained and maintained under the approval of local ethical committees.

Umbilical cords were provided by the Polish Stem Cell Bank accordingly to required approvals and procedures. UC-MSCs were isolated from five umbilical cords using an explant method as previously described [22]. Cells were cultured in DMEM/F12 (Gibco/Thermo Fisher Scientific, Waltham, MA, USA) supplemented with 10 % fetal bovine serum (FBS; Sigma-Aldrich, St. Louis, MO, USA) and penicillin/streptomycin solution (P/S; Gibco) until passages 1–2 and then they were adapted to serum-free and xeno-free media by half medium change for 2–3 days. Upon adaptation, for further experiments, cell passage was assigned as 1. Four commercially available and one previously published [23] serum-free and xeno-free media were tested along with a standard serum-based medium and a serum-reduced medium (5 % FBS) supplemented with 5 ng/mL bFGF (Peprotech, Rocky Hill, NJ, USA). A full list of media is provided in Table 1. Cells cultured in all xeno-free media were grown on surfaces coated with recombinant human fibronectin (Sigma-Aldrich, no. F1056).

**Table 1:** List of cell culture media used in the study

No.	Medium name	Company	Cat. No
M1	MSCGM-CD™ Bullet Kit	Lonza	190632
M2	PPRF—msc6—composition based on published data [23]	Sigma-Aldrich/ Gibco	[23]
M3	StemPro® MSC SFM Xenofree	Gibco	A1067501
M4	MSC NutriStem® XF Basal Medium + MSC NutriStem® XF Supplement Mix	Biological Industries (Kibbutz Beit-Haemek, Israel)	05-200-1A; 05-201-1U
M5	StemXVivo™	R&D Systems	CCM014
M6	Control-1: DMEM/F12 + 10 % FBS	Sigma-Aldrich	D6421; F0804
M7	Control-2: DMEM/F12 + 5 % FBS + bFGF (5 ng/mL)	Sigma-Aldrich/Peprotech	D6421; F0804/100-18B

HUVECs were isolated with a method described previously [24]. Briefly, umbilical vein was washed extensively with phosphate buffered saline (PBS; GE Healthcare Life Sciences HyClone Laboratories, South Logan, UT, USA) to remove blood cells, filled with 1.5 mg/mL collagenase type I solution (Sigma-Aldrich) and left for 30 min at room temperature (RT). Released cells were collected into a 50-mL tube. Additionally, the vein was perfused with three volumes of PBS and the cells were centrifuged at 300×*g* for 5 min at RT. HUVECs were cultured in EGM-2MV medium (Lonza, Basel, Switzerland) on cell culture plates coated with 0.1 % gelatin (Sigma-Aldrich).

cMSCs were isolated from heart biopsies removed during operations according to a protocol described previously [25]. cMCSs were cultured in DMEM/F12 (Sigma-Aldrich) containing 15 % FBS (Sigma-Aldrich) and P/S (Gibco).

PBMCs were isolated from peripheral blood of human healthy donors (*n* = 5) on Pancol gradient (PAN Biotech; Aidenbach, Germany). Briefly, whole blood was diluted in 1:1 ratio with PBS, gently added on the top of Pancol and centrifuged at 400×*g* for 30 min at RT. The interface containing mononuclear cells was collected and washed in five volumes of PBS, then centrifuged at 300× *g* for 7 min at RT. PBMCs were cultured in RPMI (Sigma-Aldrich) supplemented with 10 % FBS (Sigma-Aldrich) and P/S (Gibco).

### Metabolism assessment

Intracellular ATP concentration was measured with the ATPlite™ luminescence assay system (PerkinElmer, Waltham, MA, USA), according to the vendor’s recommendations. Luminescence was measured using the Infinite M200 Microplate Reader (Tecan, San Jose, CA, USA).

### Luminex-based quantitative measurement of cytokines

Conditioned media from all culture conditions were collected after the third passage and stored frozen at −80 °C prior to analysis. Concentrations of selected cytokines and chemokines were measured using the Luminex technology-based BioPlex Pro™ Human Cytokine 17-plex Assay (BioRad, Berkeley, CA, USA) and the BioPlex® MAGPIX™ Multiplex Reader (BioRad). First, media were centrifuged for 15 min at 2000×*g* to remove cell debris and then processed according to the manufacturer’s instruction. The concentrations of the following interleukins: IL-1β, IL-2, IL-4, IL-5, IL-6, IL-7, IL-8, IL-10, IL-12 (p70), IL-13, and IL-17; interferon (IFN)-γ; monocyte chemoattractant protein (MCP-1/MCAF); granulocyte colony-stimulating factor (G-CSF); macrophage colony-stimulating factor (GM-CSF); macrophage inflammatory protein (MIP-1β); and tumor necrosis factor (TNF)-α were calculated with the Bio-Plex Manager MP and Bio-Plex Manager 6.1 software (BioRad).

### Senescence assay

After the sixth passage in xeno-free and control media, cells were seeded on glass culture dishes coated with human fibronectin (Sigma-Aldrich) or without coating, respectively, and cultured for the next 3 days. Senescence assay was performed using the Senescence β-Galactosidase Staining Kit (Cell Signaling Technologies, Danvers, MA, USA), according to the manufacturer’s protocol. The senescence of the cells was assessed as the percentage of blue (β-galactosidase-positive) cells.

### Isolation of extracellular vesicles

Cell culture supernatants were collected at passages 3–4 from all tested xeno-free and control media. EVs were isolated using the sequential centrifugation protocol, as previously described [25]. Briefly, supernatants were first centrifuged at 2000×*g* for 20 min at 4 °C to remove remaining cells, cellular debris, and apoptotic bodies. Subsequently, cleared supernatants were subjected to double ultracentrifugation at 100,000×*g* for 70 min, at 4 °C, with an intermediate washing step in PBS. Obtained EVs pellets were resuspended in 150–200 μL of PBS (Lonza), and protein concentration was determined with the Bradford assay.

### Particle size analysis

The concentration and size distribution of EVs were measured with tuneable resistive pulse sensing (tRPS) technology using qNano system (Izon Science Ltd., Oxford, UK). The instrument was set up and calibrated using CPC200 beads (Izon Science) according to manufacturer’s instructions. EV samples were diluted 20× in ultrapure PBS (Lonza) and passed through a 0.45 μm Acrodisc Minispike syringe filters (Sigma-Aldrich). EVs were measured using a NP200 nanopore (analysis range 100–400 nm; Izon Science) with 20 or 10 mbar pressure. Stretch and voltage were set up in order to achieve a stable higher than 100 nA current. Samples were analyzed for 5 min or until 1000 vesicles were counted. Data processing and analysis were carried out on the Izon Control Suite software v2.2 (Izon Science).

### Western blot analysis

EV protein extracts (300 μg per medium type) were separated by Mini-PROTEAN TGXPrecast Gels (BioRad) and transferred to PVDF membranes by using Trans-Blot Turbo RTA Mini PVDF Transfer Kit (BioRad). The expression level of Syntenin and CD63 was evaluated by using goat polyclonal IgG Syntenin/SDCBP antibody (PA5-18595, Invitrogen/Thermo Fisher Scientific) and mouse monoclonal IgG exosome—anti-CD63 antibody (10628D, Invitrogen), respectively. An equal loading in the lanes was evaluated by mouse monoclonal IgG β-actin antibody (sc-81178, Santa Cruz Biotechnology). The level of analyzed proteins was subsequently detected with horseradish peroxidase (HRP)-conjugated rabbit anti-goat IgG (H+L) secondary antibody (R21459, Invitrogen) or goat anti-mouse IgG, IgM (H+L) secondary antibody (31,444, Invitrogen). All antibodies were used according to manufacturer’s protocols. The membranes were developed with Luminata Crescendo Western HRP Substrate (Merck/Millipore, Darmstadt, Germany) and imaged by Gel Doc XR+ Gel Documentation System (Bio-Rad).

### Flow cytometry

Surface antigens on UC-MSCs were analyzed after passage 3 in each of the tested media. Cells were harvested with TrypLe Select Enzyme (Gibco), and the cell suspension was centrifuged at 200×*g* for 5 min. Cells (10^5^) were stained with a specific fluorescent-conjugated antibody directed toward antigens: CD90, CD166, CD105, CD44, CD73, and HLA-DR (all PE-labeled from Biolegend, San Diego, CA, USA); CD29 (PE-Cy-5; Biolegend); or CD34, CD45, CD14, and CD16 (FITC labeled from BD Biosciences, San Jose, CA, USA). The labeling procedure was performed in 100 μL of staining buffer, composed of PBS containing 2 % FBS, for 30 min at 4 °C in the dark. Next, cells were washed in PBS, resuspended in 300 μL of staining buffer and were collected using the BD LSRFortessa flow cytometer (BD Biosciences). Obtained data were analyzed with FlowJo software (FlowJo, LLC, Ashland, OR, USA).

Surface markers on UC-MSC-derived EVs were detected using the Apogee A50-Micro Flow Cytometer (Apogee Flow Systems, Hemel Hempstead, UK) upon staining with the SYTO® RNASelect™ Green Fluorescent cell Stain solution (Molecular Probes/Thermo Fisher Scientific), a highly selective RNA dye and a subset of antibodies described above with addition of anti-CD49e antibody (Biolegend). Briefly, SYTO® RNASelect™ green fluorescent cell stain was diluted in PBS (Lonza) according to the manufacturer’s protocol and then centrifuged for 20 min at 21,000×*g* at 4 °C in order to remove dye aggregates. EVs suspended in PBS (Lonza) were incubated with the RNA dye and selected antibodies for 15 min in the dark. The percentage of positive events was calculated using Apogee Histogram software (Apogee Flow Systems).

### Quantitative real time RT-PCR analysis

For mRNA analysis, total cellular RNA was isolated using the GeneMATRIX Universal RNA Purification Kit (Eurx, Gdansk, Poland) including a DNA digestion step with the Turbo DNAse (Ambion/Thermo Fisher Scientific) and reverse transcribed with the NG dART RT Kit (Eurx) according to the manufacturer’s recommendation.

For miRNA analysis, cellular RNA was isolated with the Total RNA isolation Kit (Exiqon, Vedbaek, Denmark) with the use of Turbo DNAse (Ambion) and transcribed to complementary DNA (cDNA) with the Universal cDNA Synthesis Kit II (Exiqon) following the vendor’s recommendation.

Transcript levels were measured using the real-time PCR method with the SYBR Green Master Mix (Applied Biosystems/Thermo Fisher Scientific) and specific primer sets (Supplementary Table S1). miRNA expression was analyzed with custom-designed plates containing the miRNA locked nucleic acid (LNA)™ primers (Exiqon). Quantification of mRNA/miRNA content was performed on the 7500Fast Real-Time PCR System (Applied Biosystems) using the ΔΔCt method. Gene expression levels were calibrated with a housekeeping gene—β-2-microglobulin. miRNA content in EVs was standarized with has-miR-103-3p, identified as a normalizer by the Norm Finder tool from the GenEx software (Exiqon).

### Cell proliferation

UC-MSCs cultured in five different xeno-free media and two control media were seeded at passages 1, 3, and 6 on 24-well plates at 1 × 10^4^ cells/well in duplicate. After 3 days, cells were harvested with TrypLe Select Enzyme (Gibco) and counted using a hemocytometer.

Proliferation of cMSCs treated with UC-MSC-EVs was measured using the Cell Counting Kit-8 (Sigma-Aldrich), according to the manufacturer’s instruction. Briefly, cMSCs were seeded on 96-well plates at 10^3^ cells/well and were treated with 1 μg of EVs, which corresponded to the EV yield harvested from approximately 1.2 × 10^5^ cells, for 24 h. Then, EVs were removed and cells were kept either in hypoxia (1 % O_2_) or at normal oxygen level (21 % O_2_) for 4 days. The absorbance of formazan dye produced by living cells was measured in an Infinite M200 Microplate Reader (Tecan).

### Differentiation assays

UC-MSC cultured in xeno-free and control media were subjected to differentiation into osteocytes, chondrocytes, and adipocytes. After the third passage, 3 × 10^3^ cells were seeded on 12-well plates and were left to grow for the next 2 days before medium change into differentiation medium. StemPro® Osteogenesis Differentiation Kit, StemPro® Adipogenesis Differentiation Kit, and StemPro® Chondrogenesis Differentiation Kit (all from Gibco) were used for osteogenesis, adipogenesis, and chondrogenesis, respectively. Cells were maintained in differentiation media for 21 days, with medium change every 3–4 days. Next, cells were fixed and stained with 2 % Alizarin Red (Sigma-Aldrich) to detect calcium phosphate deposits released by osteocytes or were stained with 1 % Alcian Blue solution (Sigma-Aldrich) in order to indicate synthesis of proteoglycans by chondrocytes. Adipogenic differentiation was evaluated by the presence of fat droplets.

Cardiomyocyte differentiation of cMSCs treated with UC-MSC-EVs was performed using a method described previously [26]. Briefly, 2 × 10^4^ cMSCs were seeded per well in 12-well plates coated with 50 μg/mL collagen type I (Sigma-Aldrich) in DMEM/F12 with 15 % FBS (Sigma-Aldrich). After 24 h, UC-MSC-EVs collected from approximately 3.6 × 10^6^ cells were added (equivalence of 30 μg EVs/well) and incubated with cells for 24 h. After washing with PBS, the medium was changed into differentiation medium, composed of DMEM/F12 supplemented with 2 % FBS and 10 ng/mL bFGF, 10 ng/mL VEGF, and 10 ng/mL TGFβ1 (all growth factors from Peprotech). The medium was changed every day and after 7 days, cells were examined for cardiac differentiation.

### Immunocytochemistry

cMSCs treated with UC-MSC-EVs and subjected to cardiac differentiation were examined for expression of cardiac markers by immunocytochemistry. First, cells were fixed in 4 % paraformaldehyde, permeabilized in 0.2 % Triton X-100, and blocked with 1 % BSA. Subsequently, cells were incubated with a mouse monoclonal antibody against human GATA-4 (G-4) (Santa Cruz Biotechnology, Dallas, TX, USA) and goat polyclonal antibody against human Troponin T-C (C-19) (Santa Cruz Biotechnology) in blocking solution, containing auto MACS running buffer (Miltenyi Biotec, Bergisch Gladbach, Germany), 2 % FBS, and 2 % donkey serum. After overnight incubation, cells were rinsed and incubated with secondary antibodies Alexa Fluor 488 AffiniPure F(ab’) Fragment, donkey anti-mouse IgG (H+L) (Jackson ImmunoResearch Laboratories, West Grove, PA, USA) and Alexa Fluor® 546 donkey anti-goat IgG (H+L) (Molecular Probes) in blocking solution for 2 h. Stained cells were visualized by Leica DMI6000B ver. AF7000 microscope. Prior to analysis, nuclei were stained with Hoechst 33342 (Sigma-Aldrich).

### Capillary-like tube formation assay

HUVECs were seeded at a density of 5 × 10^4^ cells/well in the EGM-2EV medium (Lonza) on 24-well plates coated with Matrigel Matrix Grow Factor Reduced (BD Pharmingen; 100 μL/well, 30 min at RT). Fifty micrograms of EVs (harvested from approximately 6 × 10^6^ cells) was added to selected wells, and cells were incubated for 8 h at 37 °C. HUVECs without EVs were used as a control. Tube formation was investigated every 2 h, and images were collected with a Leica DMI6000B (ver. AF7000) equipped with a CCD camera. Five randomly selected images of high-power fields for cultured cell groups in tested media at every experimental time point were included in quantitative analysis and were computed as the absolute number of capillary-like structures per experimental group.

### Inhibition of PBMC proliferation

PBMCs were stained with 25 μM carboxyfluorescein succinimidyl ester (CFSE; Molecular Probes) for 15 min at 37 °C. Subsequently, the cells were stimulated with 50 ng/mL of phorbol 12-myristate 13-acetate (PMA; Sigma-Aldrich) and 1 ng/mL ionomycin (Sigma-Aldrich) for 24 h. Unstimulated cells served as a control. After double washing in PBS, 10^5^ cells were seeded into 48-well plates in 200 μL of RPMI (Sigma-Aldrich) supplemented with 10 % FBS (Sigma-Aldrich) and P/S. Next, 100 μg of UC-MSC-EVs (harvested from approximately 12 × 10^6^ cells) was added per well and cells were co-incubated with EVs for 4 days. Inhibition of PBMC proliferation was measured on a BD LSRFortessa flow cytometer (BD Biosciences) and was calculated as the decline in the number of CFSE-positive cells using the FlowJo software (FlowJo).

### Statistical analysis

The experiments were performed using five UC-MSC lines, at least twice in duplicate. The data is shown as means ± standard deviations (SD). Statistical analyses were done with one-way ANOVA and Dunnet’s post hoc test. *p* values of less than 0.05 were considered statistically significant.

## Results

### Various xeno-free media differentially maintain UC-MSCs characteristics

**Fig. 1:**
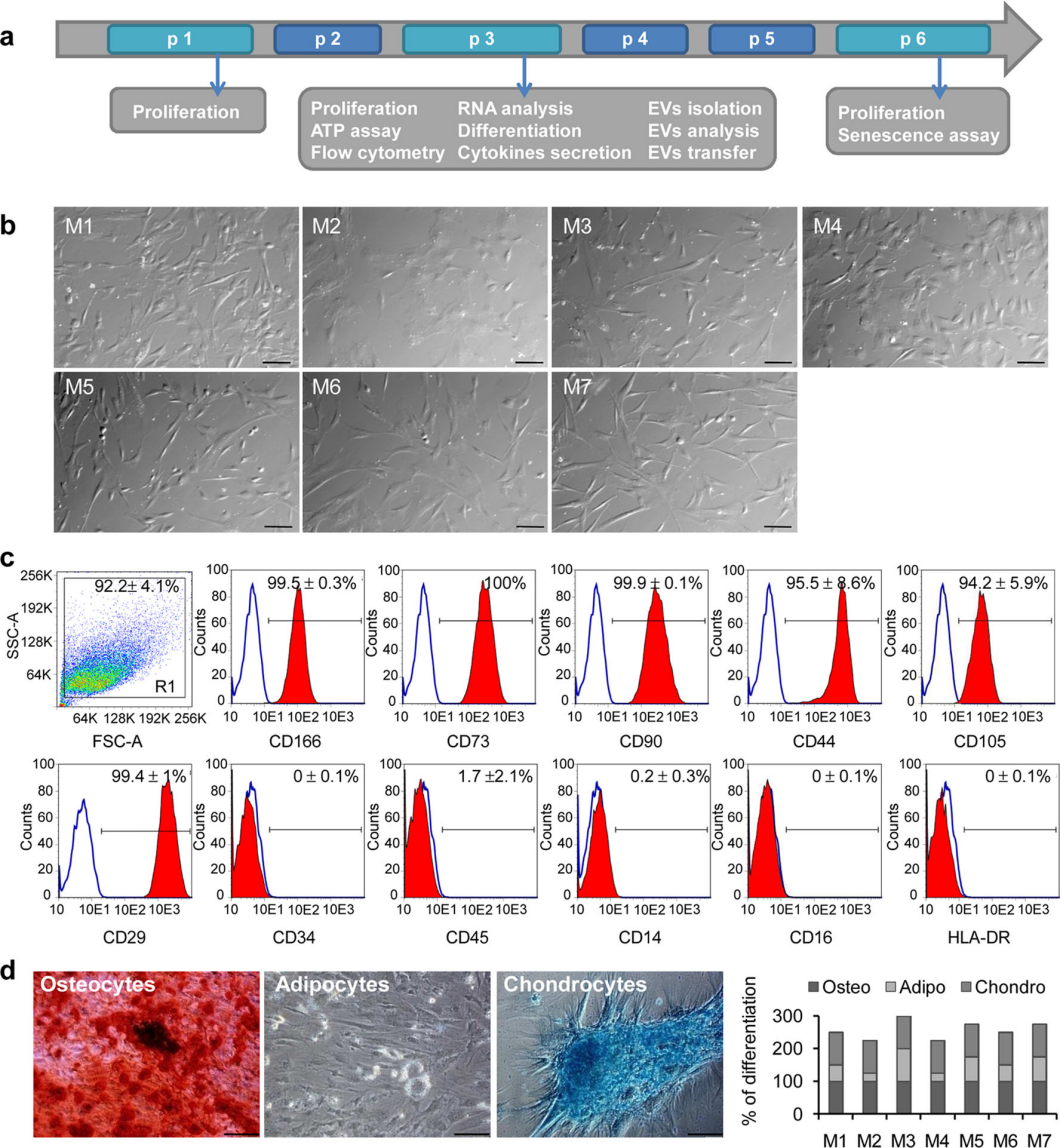
Characterization of morphology, phenotype, and differentiation potential of UC-MSCs cultured in various xeno-free media. **a** A time schedule of the study. **b** UC-MSC morphology in xeno-free (*M1*, *M2*, *M3*, *M4*, *M5*) and control media (*M6* and *M7*) at passage 1. Representative images in Nomarski Intereference Contrast are shown with a *scale bar* of 100 μm. **c** Surface antigen expression on UC-MSCs by flow cytometry. Cells were stained with selected antibodies conjugated with fluorochromes, acquired on BD LSRFortessa (BD Biosciences) and analyzed using the FlowJo software. Results are shown as a mean ± SD for cells cultured in control medium M6. Representative histograms are presented. **d** Differentiation potential of UC-MSCs into mesodermal lineages. Representative images of UC-MSCs differentiated into osteocytes, adipocytes and chondrocytes are shown as indicated (*left*). *Scale bar* shows 100 μm. Calcium phosphate deposits were stained with Alizarin Red-S, proteoglycans with Alcian Blue, and fat droplets were visible in bright field. Mean percentage of differentiation of five UC-MSCs lineages in all examined conditions (*right*). *UC-MSCs* umbilical cord-derived mesenchymal stem cells

First, we investigated the influence of various xeno-free culture conditions on UC-MSCs properties. Thus, we compared growth, phenotype, differentiation potential, metabolism, and longevity of UC-MSC cultured in xeno-free media to control cells propagated in standard serum-based medium (with 10 % FBS; M6) and serum-reduced medium (5 % FBS) supplemented with bFGF (5 ng/mL; M7), since bFGF has been shown to enhance proliferation of UC-MSCs [27]. A scheme of experimental procedures is shown in Fig. 1a.

We observed morphological differences between the groups of cells (Fig. 1b). The spindle-like shape typical for MSCs was preserved in media M1, M3, M5, and M6, with the largest longitudinal axis in medium M7. In contrast, cells were more rounded in particular in medium M2. Importantly, all the cells displayed the typical MSC phenotype with high expression of clusters of differentiation markers including CD166, CD73, CD90, CD44, CD105, and CD29 (Fig. 1c; Supplementary Table S2). Simultaneously, the cells did not express hematopoietic stem cell marker CD34, lineage commitment cell antigens (CD45, CD14, CD16), and major histocompatibility class II molecules (HLA-DR). Interestingly, the only difference in phenotype was noticed for CD105 expression, which was significantly downregulated in medium M5 and lowered in medium M2 and M3 (Supplementary Table S2).

Moreover, all the cells effectively differentiated into osteocytes and chondrocytes; however, they displayed diminished ability to differentiate into adipocytes with the exception of cells cultured in medium M3 (Fig. 1d).

**Fig. 2:**
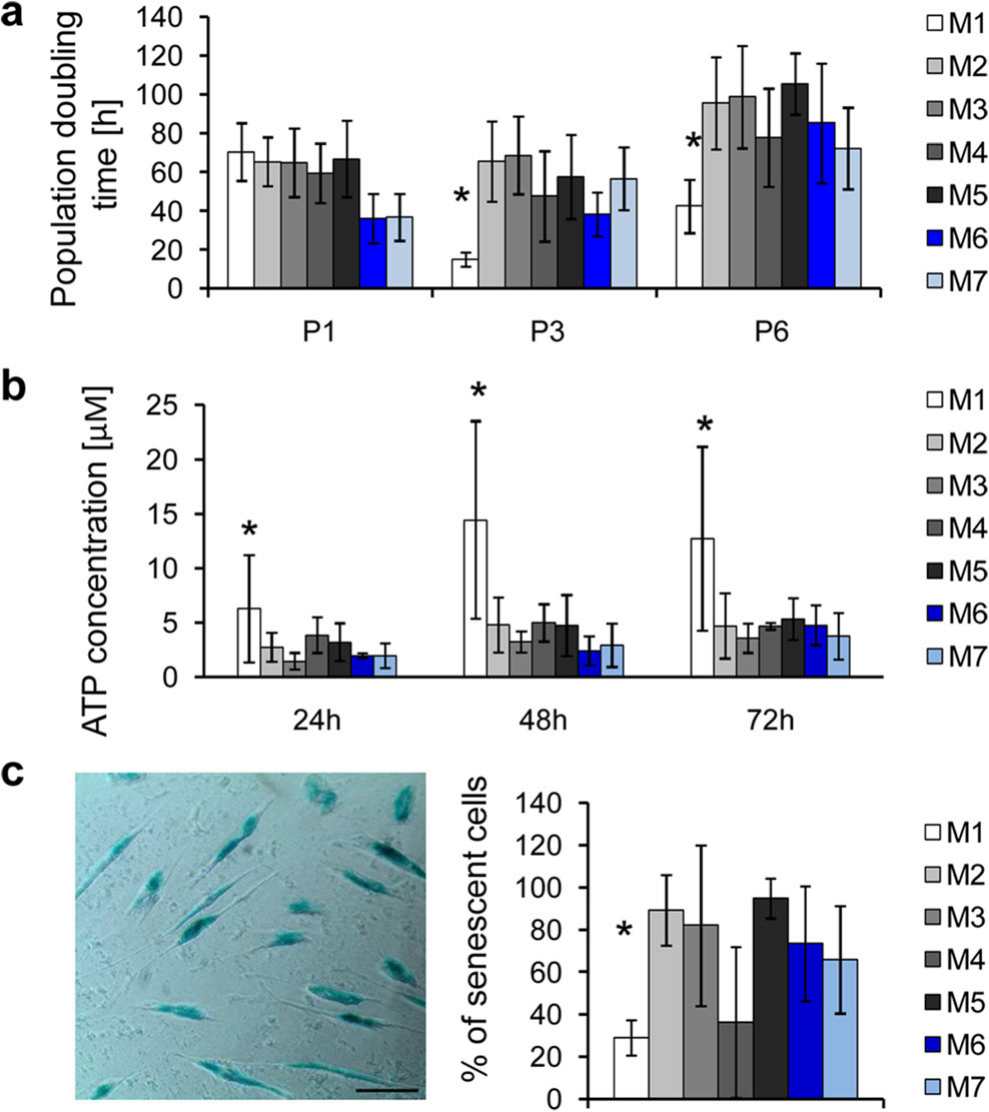
Proliferation potential, metabolic activity, and senescence rate of UC-MSCs cultured in various xeno-free conditions. **a** Proliferation rate of UC-MSCs measured after passages 1, 3, and 6 (*P1*, *P3*, *P6*) shown as population doubling time (h). Cells (10^3^) were seeded per well on 24-well plates and after 3 days the number of cells was counted in a Buerker chamber. **b** Metabolic activity of UC-MSCs. ATP concentration was evaluated 24, 48, and 72 h after seeding of 10^3^ cells/well on 96-well plates, using the ATP Assay Kit (PerkinElmer). **c** Senescence of UC-MSCs in prolonged culture. The assay was performed at passage 6 with the Senescence β-Galactosidase Staining Kit (Cell Signaling Technologies). A representative image of β-galactosidase-positive (senescent) cells with a *scale bar* of 100 μm is shown (*left*). Mean percentage of senescent cells in all examined media is shown on the graph (*right*). Results are shown as means ± SD. Statistical differences were measured with one-way ANOVA and Dunnet’s post hoc test comparing all data to control conditions (*M6*). *p < 0.05. *M1* to *M7* correspond to the tested media. *UC-MSCs* umbilical cord-derived mesenchymal stem cells

To extend characteristics beyond criteria established for MSCs by the International Society for Cellular Therapy (ISCT) [28], we also measured proliferation, metabolic, and senescence rates of UC-MSCs cultured in different conditions. The highest proliferation rate expressed as the shortest population doubling time was observed for cells cultured in medium M1 (Fig. 2a). Similarly, cells cultured in medium M1 displayed highest metabolic activity (Fig. 2b) and lowest senescence rate compared to UC-MSCs propagated in other tested conditions (Fig. 2c).

### Immunomodulatory profile of UC-MSCs is affected by cell culture conditions

**Fig. 3:**
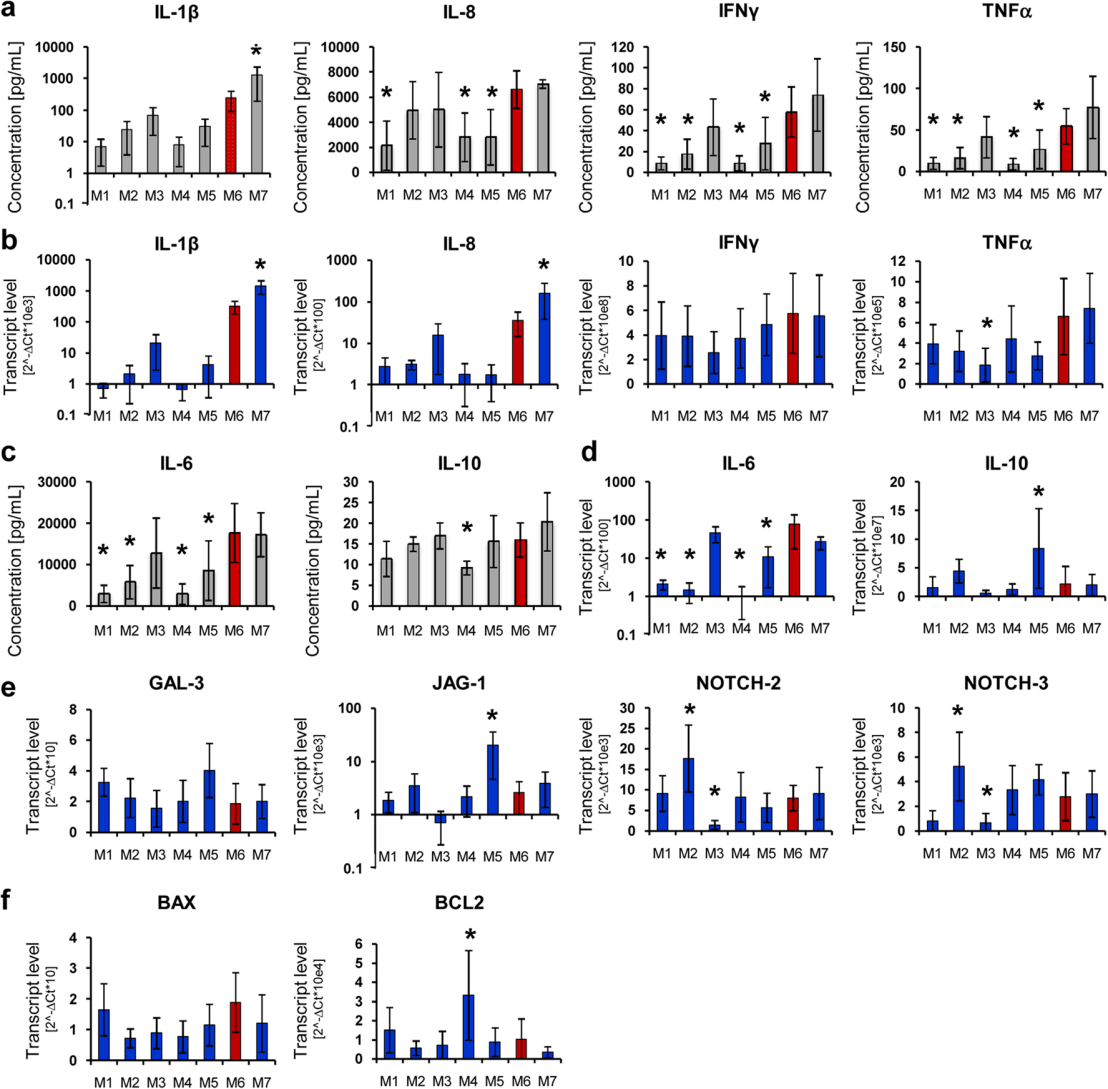
Immunomodulatory profile of UC-MSCs cultured in various xeno-free conditions. Secretion of **a** pro-inflammatory cytokines (*IL-β*, *IL-8*, *IFN-γ*, *TNF-α*) and **c** anti-inflammatory (*IL-6*, *IL-10*) cytokines was measured in conditioned media collected after passage 3 with the BioPlex Pro™ Human Cytokine 17-plex Assay (BioRad). mRNA expression levels of **b** pro-inflammatory cytokines, **d** ant-inflammatory cytokines, **e** transcripts regulating immunomodulatory ligands and receptors (*GAL-3*, *JAG-1*, *NOTCH-2*, *NOTCH-3*), and **f** apoptosis (*BAX*, *BCL2*) were measured using the real time PCR and relative quantification with the ΔΔCt method with β-2 microglobulin as endogenous control. Mean values ± SD are shown. Results were compared with one-way ANOVA and Dunnet’s post hoc test, relative to control conditions (*M6*). *p < 0.05. *M1* to *M7* correspond to the tested media. *UC-MSCs* umbilical cord-derived mesenchymal stem cells

Since UC-MSCs are known to exhibit the lowest immunogenicity compared to MSCs from other sources and are considered to be suitable for transplantation to unrelated donors, we compared the secretion of pro- and anti-inflammatory cytokines by cells cultured in different xeno-free media. In parallel, we also compared transcript levels for selected cytokines and for genes involved in immunomodulation including *GAL-3*, *JAG-1*, *NOTCH-2*, *NOTCH-3*, and regulators of apoptosis (*BAX*, *BCL-2*). The results showed that UC-MSCs cultured in media M1, M2, M4, and M5 produced lower levels of pro-inflammatory cytokines including IL-1β, IL-8, IFNγ, and TNFα compared to control medium M6 (Fig. 3a). In contrast, cells cultured in medium M7 secreted these cytokines at the highest levels, whereas medium M3 promoted cytokine production at a similar level to control medium M6 (Fig. 3a). The media themselves did not input any background into the assay and did not contain any of the measured cytokines (data not shown). The concentrations of the analyzed cytokines corresponded to their transcript levels (Fig. 3b). A similar pattern of cytokine release by UC-MSCs depending on the tested media was found for other acute-phase mediators (Supplementary Fig. S1) as well as for anti-inflammatory cytokines such as IL-6 and IL-10 (Fig. 3c), including expression of their corresponding mRNAs (Fig. 3d). Furthermore, real-time qPCR analysis revealed that the transcript level for *GALECTIN-3* (*GAL-3*)—an important regulator of immune cell activation [29]—did not differ considerably among the tested media (Fig. 3e). Interestingly, we found substantial differences in expression levels for genes related to the NOTCH signaling pathway, with the lowest mRNA levels for *NOTCH-2* and *NOTCH-3* and their ligand *JAG-1* in cells cultured in medium M3, whereas the highest *NOTCH-2* and *NOTCH-3* expression was identified in cells cultured in medium M2 (Fig. 3e). Furthermore, genes involved in regulation of apoptosis were expressed at similar levels among the different cell samples with the exception of medium M4, in which the mRNA for anti-apoptotic *BCL-2* was identified in the highest quantity (Fig. 3f).

### EVs derived from UC-MSCs cultured in various xeno-free media display different characteristics

**Fig. 4:**
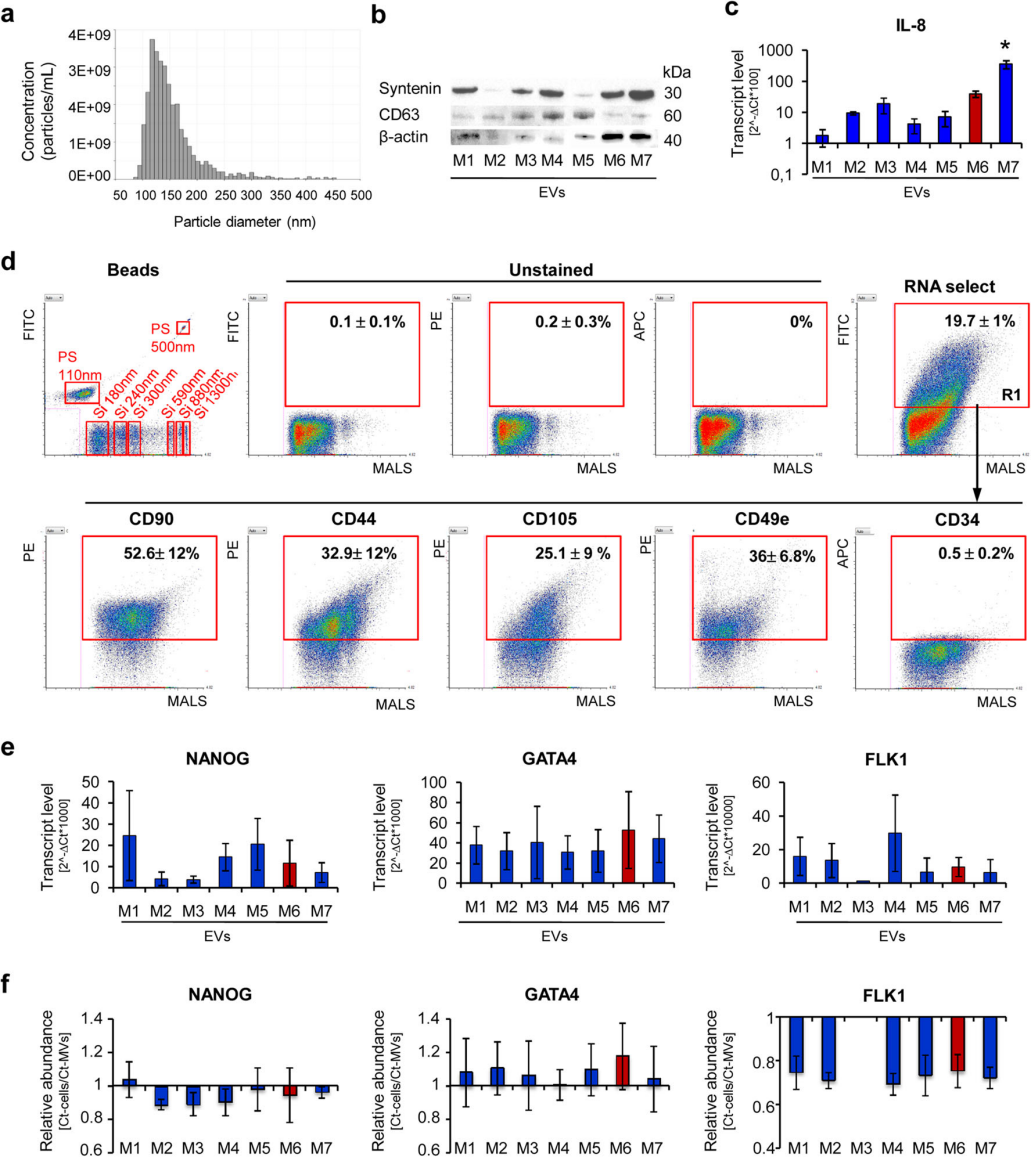
Characteristic of EVs derived from UC-MSCs cultured in xeno-free media (UC-MSC-EVs). **a** Size analysis of EVs using qNano system (Izon Science Ltd). Representative image is shown. **b** Western blot analysis of selected proteins in UC-MSC-EVs. Three hundred micrograms of protein extracts was used to detect expression of transmembrane (CD63) and cytosolic (syntenin) proteins. Expression of β-actin was used as control. **c** Transcript level for extracellular protein (IL-8) measured by RT-qPCR in UC-MSC-EVs. **d** Surface antigen profile of UC-MSC-EVs by high-sensitivity flow cytometry. The EV samples were stained with the SYTO® RNASelect™ Green Fluorescent Cell Stain (Molecular Probes) and selected antibodies labeled with a fluorochrome and further analyzed on an A50-Micro Flow Cytometer (Apogee Flow Systems). The percentage of particles positive for indicated surface marker was analyzed from SYTO® RNASelect™-positive objects (in gate R1). Representative *dot plots* for M1-EVs are shown. **e** Analysis of transcript levels for genes involved in the maintenance of pluripotency (*NANOG*) or differentiation toward cardiac (*GATA4*) and endothelial lineage (*FLK1*) performed with the real time PCR method in UC-MSC-EVs. **f** Relative transcript levels in EVs compared to parental UC-MSCs. Results are shown as mean ± SD. Results were compared with one-way ANOVA and Dunnet’s post hoc test, relative to control conditions (*M6*). *p < 0.05. *UC-MSC* umbilical cord-derived mesenchymal stem cells, *UC-MSC-EVs* extracellular vesicles derived from UC-MSCs, *M1 to M7* correspond to the tested media. *MALS* medium angle light scatter, *PS* polystyrene calibration beads, *Si* silicone calibration beads, *FITC* fluorescein isothiocyanate, *PE* phycoerythrin, *APC* allophycocyanin

Supported by the growing body of evidence that paracrine activity including cell-to-cell communication mediated by EVs plays an essential role in MSCs-mediated tissue regeneration [10–16], we examined the size, content, and biological properties of UC-MSC-EVs obtained from different conditioned media. Following isolation, general characterization of EVs was performed, according to the requirements of the International Society for Extracellular Vesicles (ISEV) [30]. Nanoparticle tracking analysis (Fig. 4a) revealed that median size of EVs collected in various xeno-free media was in a range from 134.5 ± 13.4 to 145.5 ± 13.4 nm (Supplementary Table S3) which was similar to the size of vesicles obtained in the control medium M6 (138.5 ± 3.5), with exception of M4-EVs, in which size was significantly smaller (127 ± 2.8). Next, detection of proteins associated with EVs was analyzed by western blot, indicating that all the collected EV specimens exhibited presence of tetraspanin CD63, membrane associated syntenin, although at variable levels (ISEV categories 1–2; Fig. 4b; Supplementary Fig. S2). There was no expression of an intracellular protein calnexin (ISEV category 3; data not shown), as expected. Moreover, the vesicles also contained transcripts for IL-8 (ISEV category 4; Fig. 4c) for which the highest expression was detected in case of M7-EVs. Furthermore, expression of selected surface antigens was measured on EVs using the Apogee A50-Micro flow Cytometer, a novel high-resolution platform dedicated to analysis of nanoparticles. Knowing that EV cargo comprises primarily RNAs, UC-MSC-EVs were stained with an RNA-specific dye and only RNA-containing objects were considered in analysis. The results showed that approximately 15 to 19 % of the collected particles were intact and contained RNA (Fig. 4a; Supplementary Table S4). The highest RNA content was detected in EVs derived from cells cultured in media M1 and M5 (Supplementary Table S4). Expression of surface molecules typical for MSCs, including CD90, CD44, and CD105, varied among different EV samples (Fig. 4d; Supplementary Table S4) and was highest in case of EVs harvested from media M1 and M3. Interestingly, the CD105 expression level was stable among EV samples despite differences observed in case of parental cells (Supplementary Tables S2 and S4). Moreover, all the EV samples exhibited integrin alpha-5 (CD49e; ISEV category 1; Fig. 4d); however, its highest expression was measured on M2-EVs and M3-EVs (Supplementary Table S4). The media did not generate background in the analysis since hardly any RNA-positive objects were observed in the tested media (Supplementary Fig. S3).

In order to ascertain a potential role of UC-MSC-EVs in tissue regeneration, particularly their cardiomyogenic and angiogenic potential, we analyzed mRNA content in EV specimens for several genes related to these processes. The results showed that all tested UC-MSC-EV samples contained transcripts for *NANOG*, *GATA4*, and *FLK1*, although at variable levels (Fig. 4e). Importantly, mRNA for *GATA4* was enriched in EVs compared to their parental cells (Fig. 4f). In contrast, we failed to detect mRNA for other genes including *NKX2–5* and *TIE2* in most EV samples (data not shown), although they were abundantly present in parental cells (Supplementary Fig. S4).

### Xeno-free UC-MSC-EVs enhance cardiomyogenic and angiogenic potential of cardiac and endothelial cells in vitro

To investigate potential impact of xeno-free UC-MSC-EVs on differentiation properties of target cells including their cardiomyogenic and angiogenic capacity required for heart repair, we performed functional analyses of primary heart and endothelial cells upon EVs treatment. Based on the previous data, we chose EVs derived from UC-MSCs cultured in media M1, M3, and M4 as superior for maintenance of UC-MSCs properties including proliferation rate (M1), metabolic activity (M1), longevity (M1), differentiation potential (M3), phenotype stability of the cells (M1, M4), and EVs (M1, M3), as well as low level of cytokine production (M1, M4). The biological influence of the selected types of EVs on target cells was compared to control conditions (without EVs treatment) and to the cell samples treated with control M6-EVs.

**Fig. 5:**
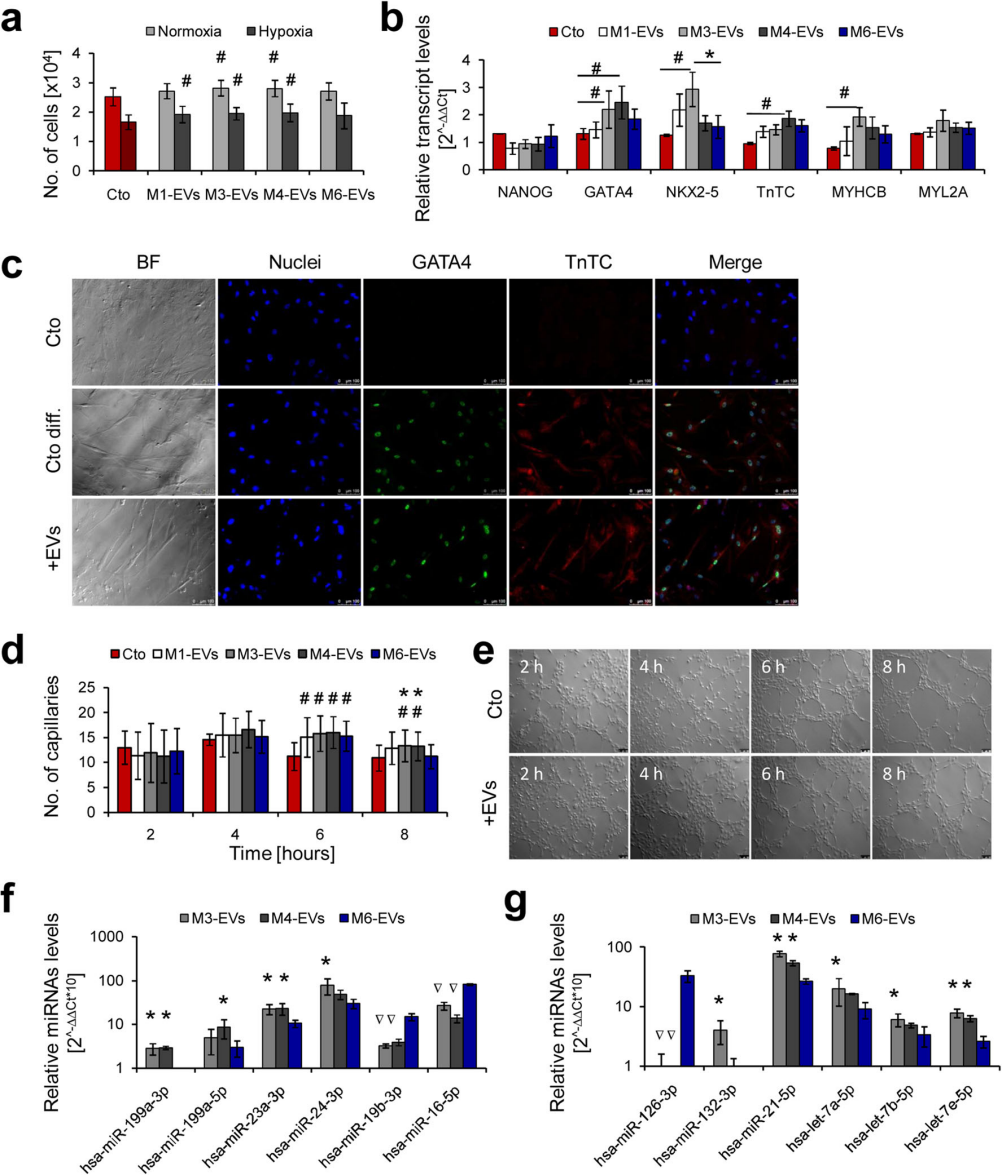
Cardiomyogenic and angiogenic potential of xeno-free UC-MCS-EVs in vitro. **a** Proliferation of cMSCs treated with UC-MSC-EVs (1 μg/10^3^ cells) and cultured 4 days in normal (21 % O_2_) or hypoxic (1 % O_2_) conditions, measured with the Cell Counting Kit-8 (Sigma-Aldrich). **b** Transcript level analysis in cMSCs treated with UC-MSC-EVs (50 μg/5 × 10^4^ cells) and subjected to cardiac differentiation for 7 days. Expression of mRNA was measured using the real time PCR and comparative ∆∆Ct analysis with β-2 microglobulin as endogenous control. **c** Immunocytochemical staining of cMSCs upon cardiac differentiation for 7 days. *Upper panel*: undifferentiated control; *middle panel*: differentiated control (cMSC without treatment with UC-MSC-EVs); *bottom panel*: cells differentiated after treatment with UC-MSC-EVs. **d** Capillary formation by human endothelial cells (HUVECs) treated with UC-MSC-EVs (50 μg/5 × 10^4^ cells). Number of capillaries was counted every 2 h during 8-h experiments in five randomly selected microscopic fields. **e** Microscopic pictures of capillaries formed by HUVECs untreated (*upper panel*) or treated with UC-MSC-EVs (*lower panel*) at indicated time points. **f** Detection of pro-cardiomyogenic miRNAs in UC-MSC-EVs. **g** Detection of pro-angiogenic miRNAs in UC-MSC-EVs. Representative images are shown for microscopic data. *Scale bars* indicate 100 μm. For *graphical charts*: results are shown as mean ± SD. Significant differences in values obtained for cells treated with UC-MSC-EVs and untreated differentiated control (#*p* < 0.05), as well as between xeno-free and control media (M6 shown as a *red column*, *p < 0.05) were evaluated by ANOVA. For miRNA analysis: samples statistically enriched or underexpressed (*p* < 0.05) compared to control M6-EVs are indicated with an *asterisk* or an *inverted triangle*, respectively. *UC-MSC-EVs* extracellular vesicles derived from umbilical cord mesenchymal stem cells, *cMSCs* cardiac mesenchymal stromal cells, *HUVECs* human umbilical vein endothelial cells, *Cto* control, *Cto diff.* differentiated control, *BF* bright field (color figure online)

First, we studied the impact of the xeno-free EVs on proliferation and cardiac differentiation of primary human cardiac cells. Upon EVs treatment, the target cells were cultured either in hypoxia (1 % O_2_) to mimic the conditions present in the ischemic heart or at normal oxygen level (21 % O_2_) and after 4 days, cell number was assessed with a cell counting kit. A significantly higher proliferation rate was observed for cMSCs treated with all three types of xeno-free EVs (M1, M3, M4) in hypoxic conditions. Moreover, EVs isolated from the conditioned media M3 and M4 increased the proliferation of cMSCs cultured in normoxia (Fig. 5a).

Cardiomyogenic differentiation of cMSCs was evaluated after 7 days following EVs treatment, based on expression levels of genes involved in cardiac development and presence of intracellular cardiac markers. The real time PCR data indicated elevated transcript levels particularly for transcription factors *GATA4* and *NKX2–5* and to a lesser extent for cardiac structural proteins—troponin TC (TnTC) and cardiac myosin heavy chain B (MYCHCB) in cells treated with M3- or M4-EVs (Fig. 5b). Results from the molecular analysis were further confirmed by immunocytochemical detection of GATA4 and TnTC proteins in the target cells (Fig. 5c).

Next, to test the angiogenic potential of selected UC-MSC-EVs, we investigated their impact on angiogenic capacity of human endothelial cells by performing capillary-like tube formation assay in vitro (Supplementary Fig. S5). Quantitative analysis revealed a significant increase in the number of capillaries formed by human endothelial cells co-incubated with all four types of EVs (including control M6-EVs) at 6 h of the experiment (Fig. 5d). This phenomenon was maintained for M3 and M4-EV-treated samples at 8 h of the assay, resulting in statistically higher number of capillaries compared to both control untreated cells and cells treated with M6-EVs (Fig. 5d). Representative images of capillary formation at every indicated time point for cells treated and untreated with EVs are shown in Fig. 5e.

### MicroRNA content of EVs determines their cardiomyogenic and angiogenic potential

We investigated the microRNA content of UC-MSC-EVs to explain their observed pro-cardiomyogenic and pro-angiogenic activity. Expression of selected miRNAs was screened in M3- and M4-EVs, which showed the most prominent stimulatory effects in both cMSCs and human endothelial cells, and was compared to the miRNA content in control M6-EVs.

The analysis revealed that UC-MSC-EVs contained various miRNAs crucial for cardiac and angiogenic cell differentiation (Fig. 5f, g). We identified significantly greater concentration of several miRNAs with pro-cardiomyogenic and cardioprotective role including miR199a-3p, miR-199a-5p [31], and miR-23a-3p [32] in M3- and M4-EVs and elevated level of miR-24a [33] in M3-EVs when compared to the control M6-EVs (Fig. 5f). Interestingly, other pro-cardiomyogenic miR-19b-3p [31] was detected at a higher level in M6-EVs (Fig. 5f). Notably, miR-16-5p which was shown to exhibit inhibitory effects on cardiomyocyte proliferation and differentiation [31] was at the same time enriched in M6-EVs (Fig. 5f). With respect to pro-angiogenic miRNAs, the analysis revealed that particularly M3-EVs contained significantly elevated levels of miR-132-3p, miR-21-5p, and miRNAs belonging to the let-7 family (let-7a-5p, let-7b-5p, and let-7f-5p) [34] compared to control (Fig. 5g). On the contrary, M6-EVs showed the highest level of miR-126 expression, one of the key regulators in angiogenesis [34] (Fig. 5g).

### UC-MSC-EVs from distinct xeno-free media possess different immunoregulatory capacity

**Fig. 6:**
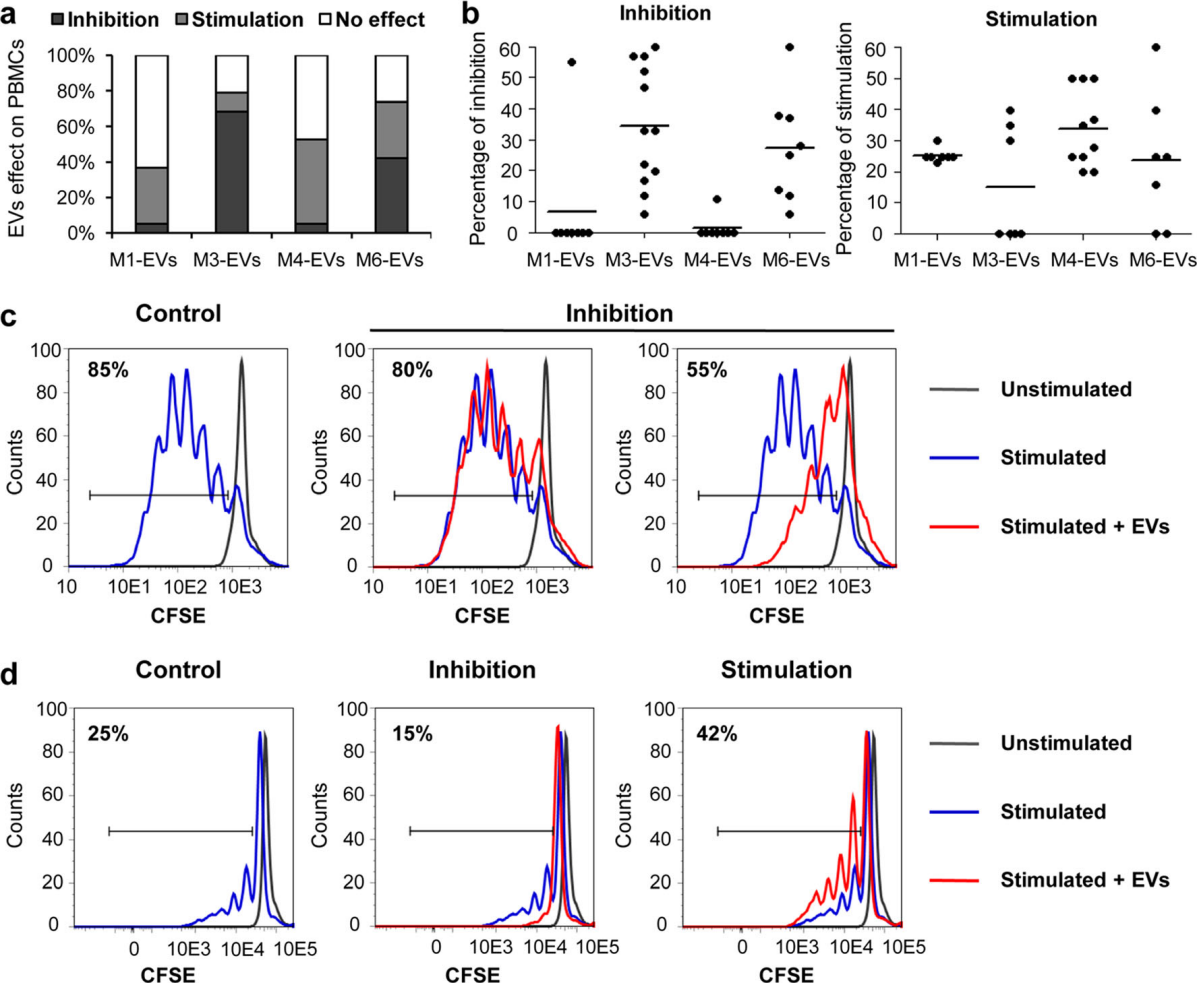
UC-MSC-EVs impact on proliferation of peripheral blood mononuclear cells (PBMCs). PBMCs were stained with 25 μM CFSE (Molecular Probes) and stimulated with 50 ng/mL PMA and 1 ng/mL ionomycin (both from Sigma-Aldrich) for 24 h. Next, cells were treated with 100 μg of UC-MSC-EVs for 4 days, and decline in CFSE-positive cells was measured by flow cytometry. **a** Summary of observed effects exerted by UC-MSC-EVs on PBMCs. Percentage of stimulation, inhibition, or no effect measured in five experiments with the use of four UC-MSC-EV types are shown. **b** Effects of single UC-MSC-EV samples on proliferation of PBMCs. Every measurement is indicated as a *black dot*, mean values are shown as *horizontal lines*. **c** Inhibition of PBMCs proliferation by two UC-MSC-EV specimens in a sample of a “good responder” to PMA/ionomycin stimulation (>80 % of PBMCs proliferation). Representative histograms are shown. **d** Dual effect of UC-MSC-EVs on PBMCs proliferation, either stimulation or inhibition, in a sample of a “poor responder” to PMA/ionomycin stimulation (<40 % of PBMCs proliferation). *UC-MSC-EVs* extracellular vesicles derived from umbilical cord mesenchymal stem cells, *PBMCs* peripheral blood mononuclear cells, *CFSE* carboxyfluorescein succinimidyl ester, *PMA* phorbol 12-myristate 13-acetate

Considering possible future clinical applications of xeno-free UC-MSC-derived EVs and their immunomodulatory properties, we performed an in vitro analysis of EVs impact on human immune cell activation. Thus, we examined proliferation status of mitogen-activated peripheral blood mononuclear cells in response to the treatment with EVs. Selected EV specimens were co-incubated with CFSE-labeled PBMCs for 4 days, and changes in CFSE-positive fraction were measured by flow cytometry. Surprisingly, the obtained data indicated distinct response of PBMCs to contact with EVs isolated from different xeno-free media. Both inhibition and stimulation of PBMCs were observed depending on the type of EVs and the initial proliferation status of the immune cells (Fig. 6). The most pronounced inhibitory effect was noticed for M3-EVs, whereas the highest rate of stimulation was observed for M4-EVs (Fig. 6a). In case of M1-EVs, the immune cells exhibited mainly no response following the treatment with EVs. The decrease or increase of PBMCs proliferation varied among the tested cell samples from 0 to 60 % (Fig. 6b). Moreover, proliferation rate of PBMCs in response to the stimulation with mitogens (PMA and ionomycin) differed among individual cell donors indicating two groups identified as “good responders” and “poor responders.” Stronger inhibition of proliferation mediated by UC-MSC-EVs was observed in the case of good responders for which the degree of proliferation was higher than 60 % (Fig. 6c). On the contrary, PBMC donors with the status of poor responders reacted to the treatment with UC-MSC-EVs either with stimulation or inhibition of proliferation (Fig. 6d).

## Discussion

Several attempts have already been made to analyze the influence of xeno-free cell culture conditions on the properties of MSCs. Growing evidence indicate similar or superior proliferative capacity as well as maintenance of three-lineage differentiation ability and surface antigen expression of MSCs cultured in xeno-free media compared to standard serum-based conditions [18–21], which was further supported by our results. However, the potential impact of such xeno-free media on the properties of MSC-EVs has not been studied.

We hypothesized that similarly to the changes triggered in cells, variable cell culture conditions may alter biological cargo within MSC-EVs, thereby promoting their specific properties. Thus, we investigated differences in characteristic of UC-MSCs cultured in various xeno-free media, as well as in therapeutic properties of extracellular vesicles derived from these cells in the context of their potential use in regenerative medicine. We particularly focused on pro-cardiomyogenic and pro-angiogenic properties of the UC-MSC-EVs, as well as their immunomodulatory function, which all may contribute to the repair of injured heart tissue [1, 35]. To address these points, we performed a comprehensive analysis of morphological, functional, and molecular features of the parental UC-MSCs and their EVs released in different culture conditions. We provide, for the first time, a versatile survey on xeno-free UC-MSC-EVs, with the goal to employ these data in potential clinical applications in the future.

**Fig. 7:**
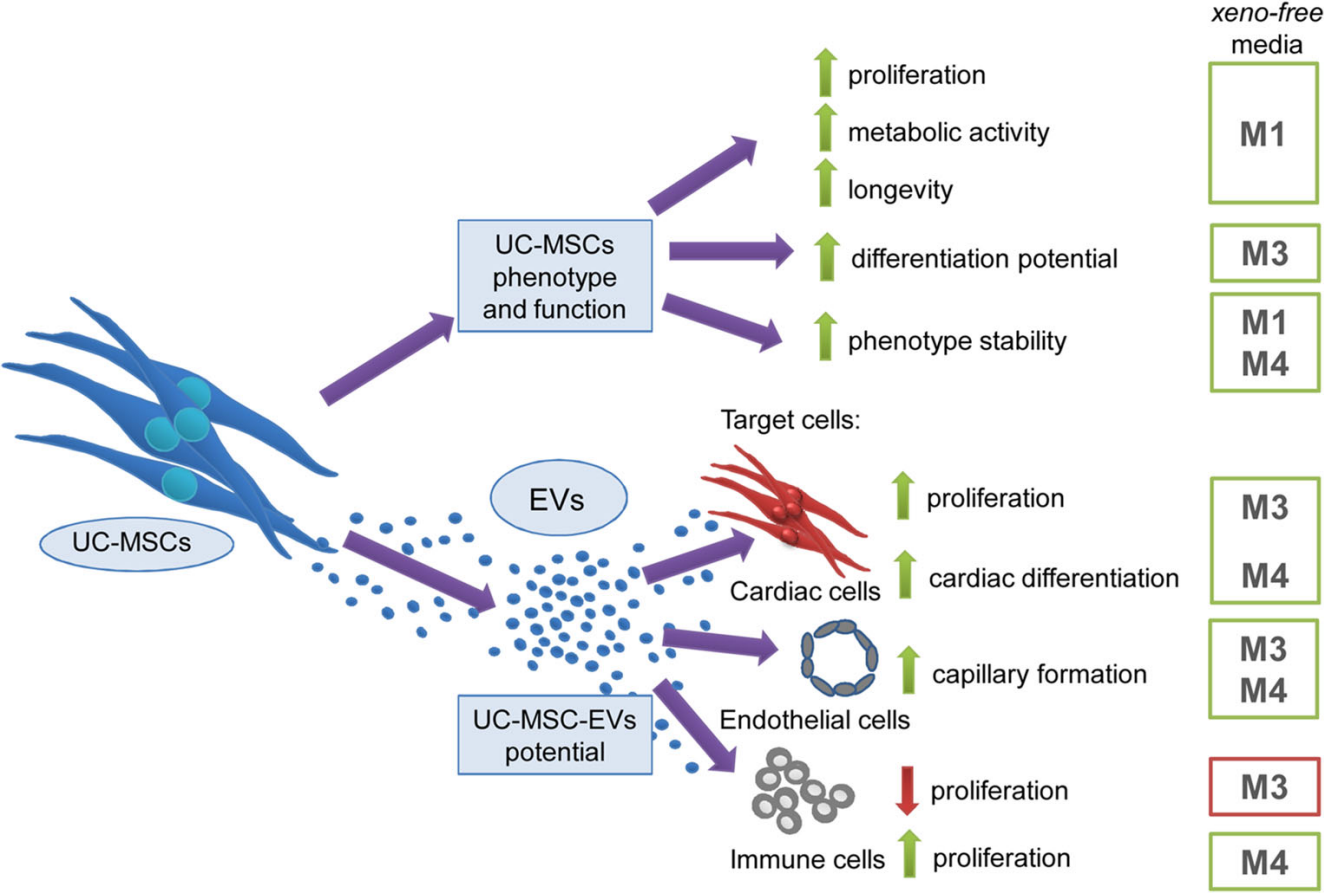
Summary of observed impact of various xeno-free media on UC-MSC and EV properties. *Upper arm*: impact of distinct xeno-free media on proliferation rate, metabolic activity, longevity, differentiation potential and phenotype stability of UC-MSCs. *Lower arm*: functional properties of EVs derived from UC-MSCs (UC-MSC-EVs) in different xeno-free media and their impact on (i) proliferation and differentiation capacity of primary cardiac cells, (ii) angiogenic capacity of endothelial cells, and (iii) proliferation of immune cells. *Green arrow* indicates stimulation/enhancement of the indicated process, while the *red arrow* shows inhibition (color figure online)

We demonstrated that cellular properties typical for MSCs including proliferation rate, metabolism, longevity, differentiation potential, and phenotype stability varied in different xeno-free media (Fig. 7, upper part). In particular, we observed that the xeno-free medium M1 was the most effective in promoting UC-MSCs proliferation, metabolic activity, and preserving their longevity, whereas medium M3 ensured three-lineage differentiation potential. Furthermore, the surface antigen profile was maintained in the xeno-free media M1 and M4.

Similarly to the influence on cellular characteristics of UC-MSCs, variable cell culture conditions impacted the characteristic and biological effects exerted by UC-MSC-EVs on target cells (Fig. 7, lower part).

In particular, medium M4 promoted secretion of EVs by UC-MSC with smallest size. Notably, M2-EVs and M5-EVs contained the membrane-bound protein syntenin at the lowest level. Similarly, expression of a cytokine, IL-8 differed among the tested samples, and its highest level was detected for M7-EVs.

Additionally, our data on cardiomyogenic activity of UC-MSC-EVs revealed that certain populations of the xeno-free EVs (M3-EVs, M4-EVs) significantly enhanced the cardiac differentiation ability of human primary heart cells in vitro. We speculate that this effect might have been triggered by RNA molecules contained in the EVs, since elevated transcript levels for pro-cardiomyogenic gene *GATA4*, as well as for certain cardiac regulatory miRNAs (miR-23a-3p, miR-24a-3p, miR-199a-3p/5p) [31–34] were detected in UC-MSC-EVs. These results are in line with data presented by Yu B et al., who showed that exosomes derived from GATA-4 overexpressing rat MSCs exerted a cardioprotective role on infarcted hearts, mainly by the activity of several cytoprotective miRNAs [36].

Importantly, MSC-EVs were already demonstrated to possess pro-angiogenic activity in vitro and in vivo [37–39]. However, differences in this potential resulting from the type of cell culture media have not been investigated so far. Our functional assay performed on human endothelial cells indicated that certain populations of the xeno-free UC-MSC-EVs (in particular M3-EVs and M4-EVs) stimulated formation of capillary-like structures in vitro more efficiently than the control M6-EVs. Thus, we speculate that application of M3-EVs or M4-EVs into an ischemic tissue may result in increased vascularization. Moreover, we found several miRNAs of known pro-angiogenic activity, including miR-132-3p, miR-21-5p, and the members of let-7 family [34], to be enriched in the xeno-free EVs compared to the control M6-EVs. On the contrary, there was no major difference in the presence of several surface receptors in the membrane compartment of these EV specimens. These results suggest that the cytoplasmic cargo rather than surface molecules defines the biological potential of the EVs.

Another important aspect of the potential utility of UC-MSC-EVs in clinical applications involves the immune response of a host toward transplanted material. It has been well documented that MSCs exert an immunosuppressive effect on various populations of immune cells, including T- and B-lymphocytes, natural killer cells, and dendritic cells [40]. Among MSCs isolated from different sources, UC-MSCs are believed to be the least immunogenic [41]. The immunomodulatory activity of MSCs is attributed to the secretion of certain mediators including IL-6, IL-10, prostaglandin E2 (PGE2), HGF, indoleamine 2,3-dioxygenase (IDO), nitric oxide (NO) TGF-b1, human leukocyte antigen G (HLA-G), and recently appreciated extracellular vesicles [40].

With respect to the immunomodulatory function of MSC-EVs, the literature shows contradictory results. Bone marrow or adipose tissue MSC-EVs were demonstrated to posses impaired ability to suppress lymphocyte proliferation, in contrast to their cellular counterparts [42, 43]. On the other hand, Zhang et al. provided evidence that exosomes secreted by human embryonic stem cell (ESC)-derived MSCs are immunologically active [44]. Despite the differences related to the source of parental MSCs, our data indicate that certain populations of MSC-EVs collected from individual donors may exert inhibitory or stimulatory effects on immune cells, depending on the type of medium used for culture of parental MSCs. In our experimental setting, M3-EVs were particularly potent inhibitors of proliferating PBMCs, in contrast to M1-EVs and M4-EVs. To explain the observed results, we provide molecular evidence of immunosuppressive character of the respective parental cells. First, we detected IL-6, suggested as a surrogate of immunosuppressive effects of stem cells [45], secreted at a high level by MSCs cultured in the medium M3 compared to other xeno-free media. Second, we found that these cells also secreted IL-10, a regulator of T-cells and a suppressor of the inflammatory immune response [46]. Furthermore, we detected a high level of expression of *Galectin-3*, which has been proposed as a biomarker for the immunomodulatory properties of UC-MSCs [47], although a similar expression pattern was found among all tested MSC populations. On the contrary, transcripts related to the NOTCH signaling pathway implicated in immune regulation by MSCs [48, 49] were present at variable levels in the tested UC-MSC samples. Surprisingly, *NOTCH-2* and *NOTCH-3* and the ligand *Jagged-1* were expressed at the lowest level by MSCs cultured in the medium M3, which suggests that an unknown mechanism was involved in promoting the immunosuppressive character of M3-EVs, which is in need of further exploration.

In summary, data obtained in this work implicate beneficial effects of certain xeno-free UC-MSC-EVs on cardiomyogenesis, angiogenesis, and immunomodulation and also highlight the importance of circumspect selection of the type of media for EVs collection to promote their specific required characteristics. In particular, we found that proliferation and cardiac differentiation of human cardiac cells as well as capillary formation by endothelial cells were stimulated to the highest degree by EVs obtained from media M3 and M4. However, the inhibition of mitogen-stimulated PBMCs was most achieved after treatment with M3-EVs, whereas the opposite effect was observed in the presence of M4-EVs (Fig. 7).

Moreover, growing evidence from incoming studies brings us closer to novel cell-free therapies with stem cell-derived specimens that may be utilized in tissue repair and other treatments in humans. Recently, the first report of successful use of MSC-EVs in the treatment of graft-versus-host disease in a human patient was published [50]. Such data make the idea of potential applications of extracellular vesicles in regenerative medicine more appealing and bring it closer to the clinical trials in the near future. Results from our study further support this concept showing a novel, effective cell-free treatment option, which may be customized for certain applications by selecting an appropriate xeno-free cell culture system for UC-MSCs propagation and EVs collection.

## Electronic supplementary material

ESM 1 (PDF 403 kb)
