# Supplementary material for: Diverse impact of xeno-free conditions on biological and regenerative properties of hUC-MSCs and their extracellular vesicles
Source: J Mol Med (Berl). 2016 Sep 16;95(2):205–20. doi: 10.1007/s00109-016-1471-7 (PMC5239805; doi:10.1007/s00109-016-1471-7)
Supplement: Supplementary file 1 — (PDF 403 kb) [file 109_2016_1471_MOESM1_ESM.pdf]

# Electronic supplementary material (ESM)

***J Mol Med 2016***

Title: **Diverse impact of xeno-free conditions on biological and regenerative properties of hUC-MSCs and their extracellular vesicles**

Sylwia Bobis-Wozowicz[1]*#, Katarzyna Kmiotek[1]*, Karolina Kania[1], Elzbieta Karnas[1,2], Anna Labedz-Maslowska[1], Malgorzata Sekula[2], Sylwia Kedracka-Krok[3], Jacek Kolcz[4], Dariusz Boruczkowski[5], Zbigniew Madeja[1], Ewa K Zuba-Surma[1]#.

[1]Department of Cell Biology, Faculty of Biochemistry, Biophysics and Biotechnology, Jagiellonian University, Krakow, 30-387, Poland

[2]Malopolska Centre of Biotechnology, Krakow, 30-387, Poland

[3]Department of Physical Biochemistry, Faculty of Biochemistry, Biophysics and Biotechnology, Jagiellonian University, Krakow, 30-387, Poland

[4]Department of Pediatric Cardiac Surgery, Polish-American Children's Hospital, Krakow, 30-663, Poland

[5]Polish Stem Cell Bank, 00-131, Warsaw, Poland

*These authors equally contributed to the work

#Corresponding authors:

Ewa Zuba-Surma
ewa.zuba-surma@uj.edu.pl
Tel. (+48) 12 664 6180
Fax. (+48) 12 664 6902

Sylwia Bobis-Wozowicz
sylwia.bobis@uj.edu.pl
Tel. (+48) 12 664 6227
Fax. (+48) 12 664 6902

## Supplementary materials

**Table S1. List of qPCR primers (all for human sequences).**

| No. | Gene Name | Gene symbol | Forward (5' – 3') | Reverse (5' – 3') | Ref. |
|---|---|---|---|---|---|
| 1 | Nanog homeobox | NANOG | ACCTCAGCTACAAACAGGTGAAG | TTCTGCGTCACACCATTGCT | [1] |
| 2 | GATA binding protein 4 | GATA4 | AACGACGGCAACAACGATAAT | GTTTTTTCCCCTTTGATTTTTGATC | [2] |
| 3 | NK2 transcription factor-related, locus 5 | NKX2-5 | CCCCTGGATTTTGCATTCAC | CGTGCGCAAGAACAAACG | [2] |
| 4 | troponin T type 2 cardiac | TnTC | ATGAGCGGGAGAAGGAGCGGCAGAAC | TCAATGGCCAGCACCTTCCTCCTCTC | [3] |
| 5 | myosin, heavy chain 7, cardiac muscle, beta | MYHCB | CTGGAGGCCGAGCAGAAGCGCAACG | GTCCGCCCGCTCCTCTGCCTCATCC | [3] |
| 6 | myosin, light chain 7, regulatory | MYL2A | GGGCCCCATCAACTTCACCGTCTTCC | TGTAGTCGATGTTCCCCGCCAGGTCC | [3] |
| 7 | kinase insert domain receptor | FLK1 | GGTTGTGTATGTCCCACCCC | GAGTGGTGCCGTACTGGTAG | [1] |
| 8 | TEK tyrosine kinase, endothelial | TIE2 | TCCCGAGGTCAAGAGGTGTA | AGGGTGTGCCTCCTAAGCTA | [1] |
| 9 | interleukin 1 beta | IL-1β | AGACATCACCAAGCTTTTTTGCT | GCACGATGCACCTGTACGAT | [4] |
| 10 | interleukin 6 | IL-6 | TTCGGCAAATGTAGCATG | AATAGTGTCCTAACGCTCATAC | [5] |
| 11 | interleukin 8 | IL-8 | TTAGCACTCCTTGGCAAAACTG | CTGGCCGTGGCTCTCTTG | [4] |
| 12 | interleukin 10 | IL-10 | GTGGAGCAGGTGAAGAATGC | GCCACCCTGATGTCTCTCAGTT | [6] |
| 13 | interferon, gamma | IFNγ | GTTCCATTATCCGCTACATCTGAA | TCAGCTCTGCATCGTTTTGG | [4] |
| 14 | tumor necrosis factor alpha | TNFα | CCTCTGATGGCACCACCAG | TCTTCTCGAACCCCGAGTGA | [4] |
| 15 | lectin, galactoside-binding, soluble, 3 | GAL-3 | CCAAAGAGGGAATGATGTTGCC | TGATTGTACTGCAACAAGTGAGC | [7] |
| 16 | jagged 1 | JAG-1 | CGGCCTCTGAAGAACAGAAC | TCACCAAGCAACAGATCCAA | [8] |
| 17 | neurogenic locus notch homolog protein 2 | NOTCH-2 | TGGGCTACACTGGGAAAAAC | TAGGCACTGGGACTCTGCTT | [8] |
| 18 | neurogenic locus notch homolog protein 3 | NOTCH-3 | CTCATCCGAAACCGCTCTAC | TCTTCCACCATGCCCTCTAC | [8] |
| 19 | BCL2-associated X protein | BAX | TTTTGCTTCAGGGTTTCATCCAG | CGGAAAAAGACCTCTCGGGG | S.B-W |
| 20 | B-cell CLL/lymphoma 2 | BCL2 | GATAACGGAGGCTGGGATGC | TGACTTCACTTGTGGCCCAG | S.B-W |
| 21 | beta-2-microglobulin | β2m | AATGCGGCATCTTCAAAC | TGACTTTGTCACAGCCCAAGATA | [9] |

**Table S2. Phenotype of UC-MSC in various xeno-free media.** Results are presented as means ± standard deviation. Values were rounded to one decimal place. Statistically significant differences at p<0.05 in comparison to control medium (M6) are indicated with an asterisk in bold.

| | Percentage of positive cells | | | | | | |
|---|---|---|---|---|---|---|---|
| **Antigen/Medium** | **M1** | **M2** | **M3** | **M4** | **M5** | **M6** | **M7** |
| **CD166** | 99.4 ± 0.2 | 92 ± 10.8 | 94.4 ± 6.8 | 99.6 ± 0.5 | 92.2 ± 8.9 | 99.5 ± 0.3 | 95.3 ± 4.3 |
| **CD73** | 99.6 ± 2.2 | 93.4 ± 7.7 | 98.9 ± 1.1 | 99.4 ± 0.9 | 98.4 ± 2.2 | 100 | 99.7 ± 0.3 |
| **CD90** | 99.2 ± 0.1 | 94.9 ± 8.5 | 99.8 ± 0.2 | 99.6 ± 0.3 | 97 ± 4.7 | 99.9 ± 0.1 | 97.8 ± 2 |
| **CD44** | 99.2 ± 0.1 | 87.4 ± 13 | 99.2 ± 0.7 | 99.8 ± 0.2 | 99.3 ± 0.3 | 95.5 ± 8.6 | 96.4 ± 6.3 |
| **CD105** | 91.8 ± 1.2 | 83.6 ± 7.6 | 81.1 ± 14 | 92 ± 6.1 | **65 ± 24*** | 94.2 ± 5.9 | 91.2 ± 4.1 |
| **CD29** | 97.8 ± 3.7 | 90.4 ± 11 | 99.5 ± 0.6 | 99.9 ± 0.1 | 97.3 ± 4 | 99.4 ± 1 | 99.8 ± 0.2 |
| **CD34** | 0 ± 0.3 | 2.6 ± 5.8 | 0 ± 0.1 | 0 ± 0.1 | 0.2 ± 0.2 | 0 ± 0.1 | 0 ± 0.1 |
| **CD45** | 1.3 ± 2.4 | 2 ± 4.2 | 2.9 ± 4.0 | 2.3 ± 4.3 | 0.9 ± 1.7 | 1.7 ± 2.1 | 1.3 ± 1.7 |
| **CD14** | 0.1 ± 0.4 | 0.1 ± 0.1 | 0.2 ± 0.3 | 0.1 ± 0.1 | 0.3 ± 0.4 | 0.2 ± 0.3 | 0 ± 0.1 |
| **CD16** | 0.1 ± 0.3 | 0.5 ± 1.2 | 0.2 ± 0.2 | 0.1 ± 0.1 | 0.2 ± 0.3 | 0 ± 0.1 | 0.3 ± 0.2 |
| **HLA-DR** | 0.5 ± 1.3 | 0.1 ± 0.1 | 0.6 ± 0.6 | 0.4 ± 1 | 0.2 ± 0.2 | 0 ± 0.1 | 0.1 ± 0.1 |

**Table S3. Nanoparticle size analysis.** Size distribution of UC-MSC-EVs was measured with IZON (IZON Science; Cambridge, MA, USA). Mean and mode with standard deviation for each medium type are presented. Statistically significant differences at p<0.05 in comparison to control medium (M6) are indicated with an asterisk in bold.

| EV type | Mean | Median |
|---|---|---|
| **M1-EVs** | **163 ± 1.4*** | 134.5 ± 13.4 |
| **M2-EVs** | 180.5 ± 14.8 | 141 ± 7.1 |
| **M3-EVs** | 167.5 ± 17.7 | 138.5 ± 19.1 |
| **M4-EVs** | 170.5 ± 9.2 | **127 ± 2.8*** |
| **M5-EVs** | 181 ± 25.5 | 140 ± 21.2 |
| **M6-EVs** | 181± 8.5 | 138.5 ± 3.5 |
| **M7-EVs** | 181.5 ± 23.3 | 145.5 ± 13.4 |

**Table S4. Surface antigens detection on extracellular vesicles derived from UC-MSCs.** Results are presented as means ± standard deviation. Values were rounded to one decimal place. Statistically significant differences at p<0.05 in comparison to control medium (M6) are indicated with an asterisk in bold.

| | Percentage of positive events | | | | | | |
|---|---|---|---|---|---|---|---|
| **Marker/EVs** | **M1-EVs** | **M2-EVs** | **M3-EVs** | **M4-EVs** | **M5-EVs** | **M6-EVs** | **M7-EVs** |
| **RNA** | **19.7 ± 1*** | 16.3 ± 1.2 | 15.1 ± 0.5 | 16.5 ± 0.2 | **19 ± 0.5*** | 14.4 ± 0.7 | 16.5 ± 0.9 |
| **CD90** | **52.6 ± 12*** | 45.9 ± 13 | **49 ± 6.6*** | 36.5 ± 8.7 | 31.4 ± 9.5 | 30.9 ± 13 | 27.3 ± 7 |
| **CD44** | **32.9 ± 12*** | **22.8 ± 11*** | **24 ± 5.8*** | 17.5 ± 8.8 | 21.3 ± 4.5 | 8.5 ± 6 | 12.7 ± 2.9 |
| **CD105** | 25.1 ± 9 | 28.8 ± 4.4 | 25.9 ± 5 | 25 ± 3.4 | 23.1 ± 4.9 | 23.4 ± 7.4 | 26 ± 13.6 |
| **MFI-CD105** | 82 ± 6.5 | 87 ± 13 | 73 ± 12 | 89 ± 17 | 57 ± 4 | 68 ± 8.1 | 79 ± 7 |
| **CD49e** | 36 ± 6.8 | **63 ± 6.2*** | **47 ± 0.1*** | 33 ± 1.1 | 34 ± 5.5 | 33 ± 3.3 | 31 ± 8.4 |
| **CD34** | 0.5 ± 0.2 | 1.6 ± 2.3 | 0.6 ± 0.3 | 0.5 ± 0.2 | 0.3 ± 0.1 | 0.4 ± 0.1 | 0.5 ± 0.2 |

.

## Supplementary figures

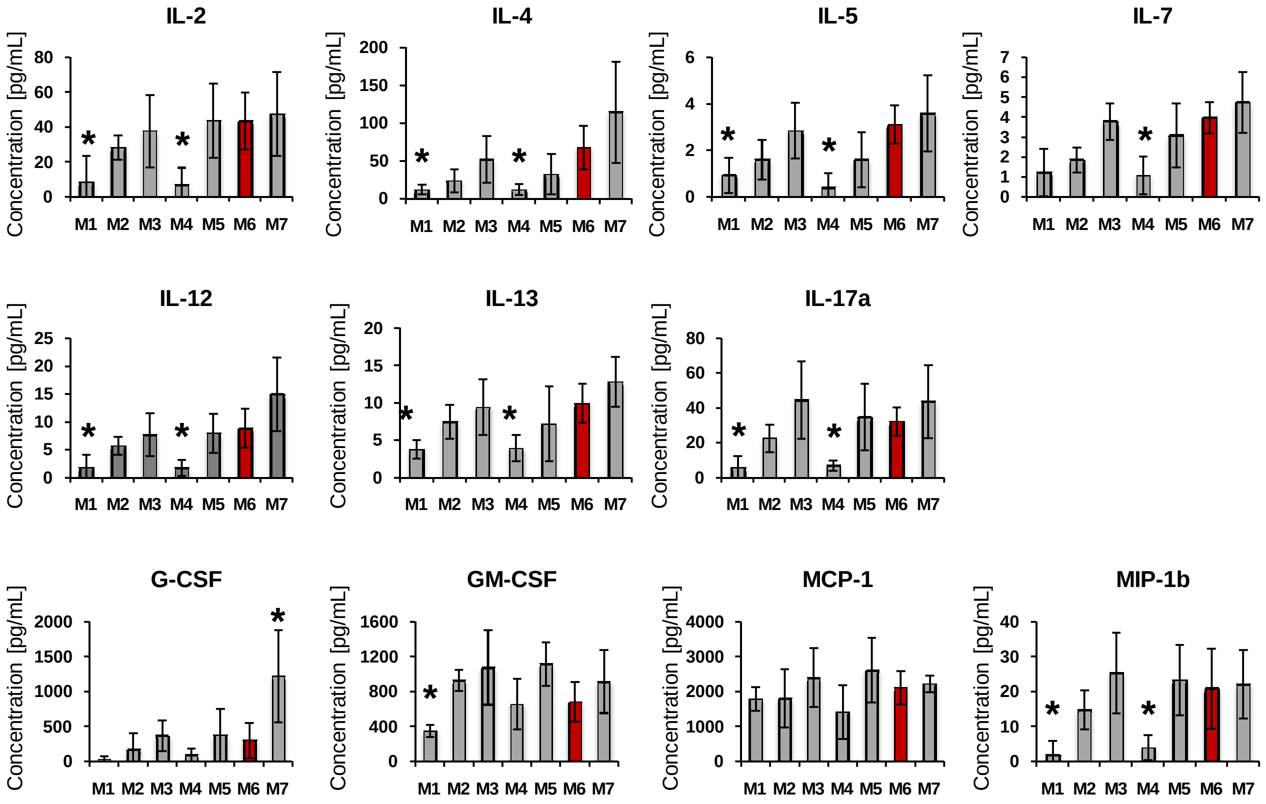


**Fig. S1.** Secretion of cytokines by UC-MSC cultured in xeno-free and standard media. Concentration of selected interleukines (IL-2, -4, -5, -7, -12, -13, -17a) and other cytokines, including granulocyte - colony stimulating factor (G-CSF), granulocyte macrophage – colony stimulating factor (GM-CSF), monocyte chemoatrractant protein –1 (MCP-1) and macrophage inflammatory protein – 1 beta (MIP-1b) was measured in conditioned media collected after passage 3 with the BioPlex Pro™ Human Cytokine 17-plex Assay (BioRad). Results were compared with one-way ANOVA and Dunnet post-hoc test to control conditions (M6). * $p<0.05$. Mean values ± standard deviations are shown. UC-MSCs - Umbilical Cord-derived Mesenchymal Stem Cells.

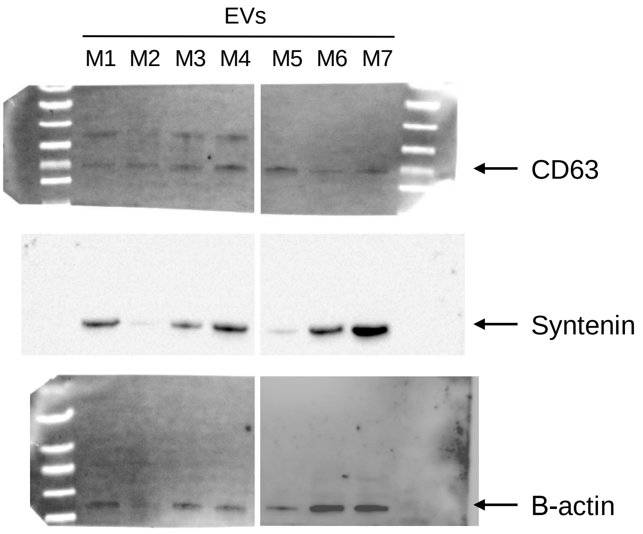


**Fig. S2.** Western blot analysis of proteins typical for EVs. Equal protein amounts (300 µg) were used for each EV type (M1-M7). Proteins were separated by PAGE and transferred to PVDF membranes to detect CD63, Syntenin and β-actin using specific primary and horseradish peroxidase (HRP)-conjugated secondary antibodies, as described in *Materials and Methods*. Original Western blot pictures are shown. EVs – Extracellular Vesicles derived from UC-MSCs; PAGE – polyacrylamide gel electrophoresis; PVDF – polyvinylidene fluoride.

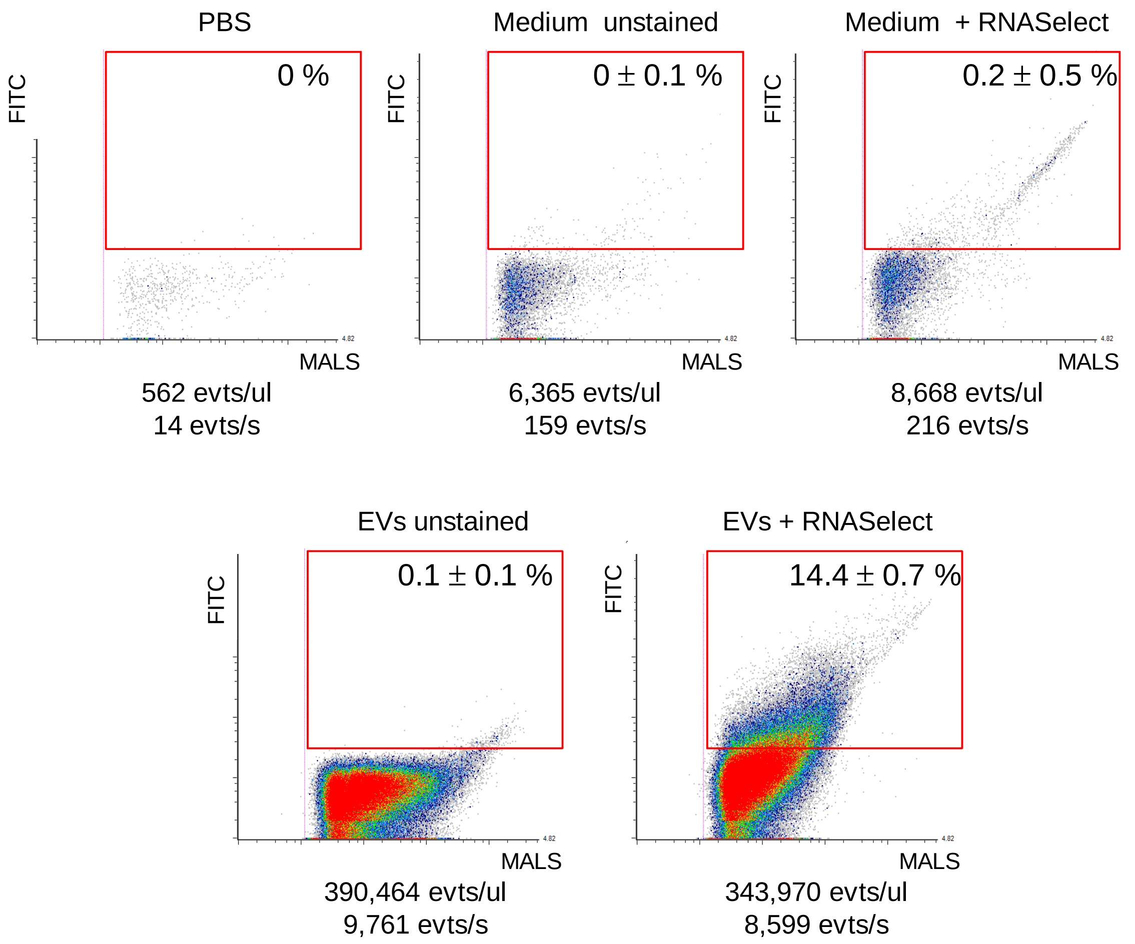


**Fig. S3.** Flow cytometry analysis of RNA-positive objects in the xeno-free and standard cell culture media used in the study. by A50-Micro Flow Cytometer (Apogee Flow Systems). The percentage of RNA-positive events in the samples were calculated considering concentration of the particles in the samples and validated on EVs sample stained with the SYTO® RNASelect™ dye. Representative dot plots are shown. PBS – phosphate buffered saline; EVs – Extracellular Vesicles derived from UC-MSCs; MALS - Medium Angle Light Scatter; FITC - fluorescein isothiocyanate;

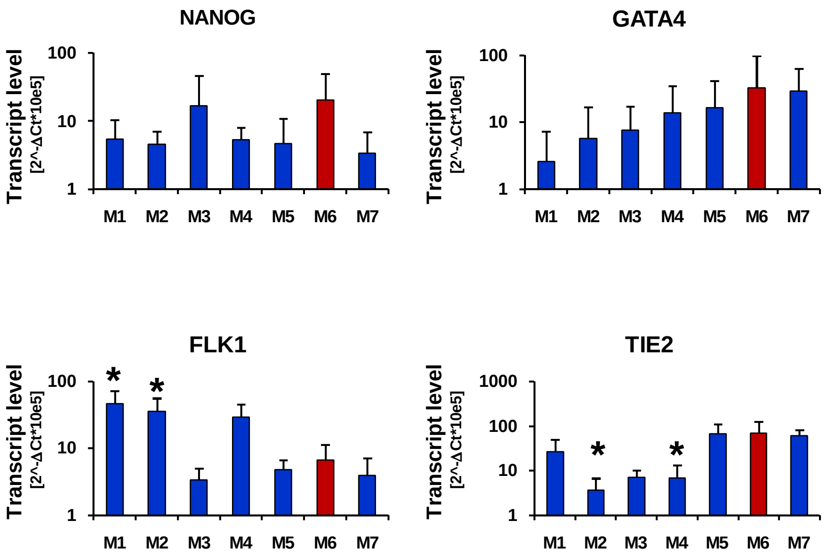


**Fig. S4**. Relative transcript levels in UC-MSCs cultured in xeno-free media and in standard conditions. The real time PCR method was employed to measure mRNA levels for genes from the pluripotency network (*NANOG*), regulators of cardiac development (*GATA4*, *NKX2.5*) and angiogenesis (*FLK1*, *TIE2*). Results were calculated with the ΔΔCt method using β2-microglobulin as endogenous control and compared with one-way ANOVA and Dunnet post-hoc test to control conditions (M6). * $p<0.05$. Mean values ± standard deviations are shown.

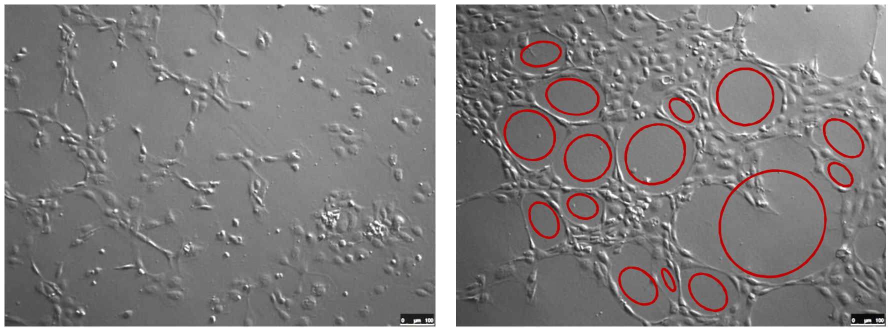

**Fig. S5.** Schematic representation of capillary-like tube enumeration. Capillary-like structures were formed by the human umbilical vein endothelial cells (HUVECs) treated or untreated with extracellular vesicles collected from the xeno-free cultures of umbilical cord-derived mesenchymal stem cells (UC-MSC-EVs). Representative images of endothelial cells directly after seeding on a matrigel (left) and after 6 hours of the assay (right) with capillaries indicated with red circles. Scale bar = 100 µm.

## References to supplementary materials:


1. Bobis-Wozowicz S, Kmiotek K, Sekula M, Kedracka-Krok S, Kamycka E, Adamiak M, Jankowska U, Madetko-Talowska A, Sarna M, Bik-Multanowski M, et al. (2015) Human Induced Pluripotent Stem Cell-Derived Microvesicles Transmit RNAs and Proteins to Recipient Mature Heart Cells Modulating Cell Fate and Behavior. Stem Cells 33:2748-2761
2. Wojakowski W, Tendera M, Michałowska A, Majka M, Kucia M, Maślankiewicz K, Wyderka R, Ochała A, Ratajczak MZ (2004) Mobilization of CD34/CXCR4+, CD34/CD117+, c-met+ stem cells, and mononuclear cells expressing early cardiac, muscle, and endothelial markers into peripheral blood in patients with acute myocardial infarction. Circulation 110:3213-3220
3. Takahashi K, Tanabe K, Ohnuki M, Narita M, Ichisaka T, Tomoda K, Yamanaka S (2007) Induction of Pluripotent Stem Cells from Adult Human Fibroblasts by Defined Factors. Cell 131:861–872
4. Ratajczak J, Kucia M, Mierzejewska K, Marlicz W, Pietrzkowski Z, Wojakowski W, Greco NJ, Tendera M, Ratajczak MZ (2013) Paracrine proangiopoietic effects of human umbilical cord blood-derived purified CD133+ cells--implications for stem cell therapies in regenerative medicine. Stem Cells Dev 22:422-430
5. Zhao X, Liu L, Liu D, Fan H, Wang Y, Hu Y, Hou Y (2012) Progesterone enhances immunoregulatory activity of human mesenchymal stem cells via PGE2 and IL-6. Am J Reprod Immunol 68:290-300
6. Favaro E, Carpanetto A, Lamorte S, Fusco A, Caorsi C, Deregibus MC, Bruno S, Amoroso A, Giovarelli M, Porta M, et al (2014) Human mesenchymal stem cell-derived microvesicles modulate T cell response to islet antigen glutamic acid decarboxylase in patients with type 1 diabetes. Diabetologia 57:1664-1673
7. Gieseke F, Böhringer J, Bussolari R, Dominici M, Handgretinger R, Müller I (2010) Human multipotent mesenchymal stromal cells use galectin-1 to inhibit immune effector cells. Blood 116:3770-3779
8. Li X, Bai J, Ji X, Li R, Xuan Y, Wang Y. Comprehensive characterization of four different populations of human mesenchymal stem cells as regards their immune properties, proliferation and differentiation (2014) Int J Mol Med 34:695-704.
